# Bcl9 and Pygo synergise downstream of *Apc* to effect intestinal neoplasia in FAP mouse models

**DOI:** 10.1038/s41467-018-08164-z

**Published:** 2019-02-13

**Authors:** Juliusz Mieszczanek, Laurens M. van Tienen, Ashraf E. K. Ibrahim, Douglas J. Winton, Mariann Bienz

**Affiliations:** 1 MRC Laboratory of Molecular Biology, Cambridge Biomedical Campus, Francis Crick Avenue, Cambridge, CB2 0QH UK; 2 Cancer Research UK Cambridge Institute, University of Cambridge, Li Ka Shing Centre,, Robinson Way, Cambridge, CB2 0RE UK

## Abstract

Bcl9 and Pygo are Wnt enhanceosome components that effect β-catenin-dependent transcription. Whether they mediate β-catenin-dependent neoplasia is unclear. Here we assess their roles in intestinal tumourigenesis initiated by Apc loss-of-function (*Apc*^*Min*^), or by *Apc*^*1322T*^ encoding a partially-functional Apc truncation commonly found in colorectal carcinomas. Intestinal deletion of Bcl9 extends disease-free survival in both models, and essentially cures *Apc*^*1322T*^ mice of their neoplasia. Loss-of-Bcl9 synergises with loss-of-Pygo to shift gene expression within *Apc*-mutant adenomas from stem cell-like to differentiation along Notch-regulated secretory lineages. Bcl9 loss also promotes tumour retention in *Apc*^*Min*^ mice, apparently via relocating nuclear β-catenin to the cell surface, but this undesirable effect is not seen in *Apc*^*1322T*^ mice whose Apc truncation retains partial function in regulating β-catenin. Our results demonstrate a key role of the Wnt enhanceosome in β-catenin-dependent intestinal tumourigenesis and reveal the potential of BCL9 as a therapeutic target during early stages of colorectal cancer.

## Abstract (web summary)

BCL9 and Pygo are components of Wnt enhanceosome, which facilitates β-catenin-dependent transcription. Here, the authors show that deletion of Bcl9 and Pygo suppresses tumorigenesis and extends disease free survival in two different colorectal cancer models, suggesting a strategy for drugging β-catenin signalling in this cancer.

## Introduction

Colorectal cancer is the second most common cause for cancer mortality in the developed world (http://globocan.iarc.fr). The pathway to this cancer is usually initiated by the mutational inactivation of the *Adenomatous Polyposis Coli* (*APC*) tumour suppressor, both in sporadic and hereditary forms^1^. Individuals bearing *APC* germline mutations develop thousands of benign adenomas in their large intestine by their teenage years one of which, invariably, progresses to carcinoma within the next decades of their lives^2^. Progression to colorectal cancer requires a small number of additional driver mutations, e.g., the activation of the KRAS or PIK3CA oncogenes, and/or the inactivation of the P53 or ARID1A tumour suppressors^3^. Notably, *Apc* germline mutations in the mouse also cause multiple intestinal neoplasia (*Min*), and various *Apc* loss-based models have been developed for pre-clinical studies^4^, whereby the original *Apc*^*Min*^ model^5^ has been used most widely.

At the molecular level, APC attenuates Wnt signal transduction through the ‘canonical’ branch, by cooperating with Axin to promote the proteasomal degradation of the key effector β-catenin. This process is inhibited by Wnt signals that promote the stabilisation of β-catenin, allowing it to access T cell factors (TCF) bound to *cis*-regulatory enhancers of Wnt-responsive genes to operate Wnt-dependent transcription. Key to this process is the Wnt enhanceosome^6,7^, a multiprotein complex tethered via TCF to enhancers of lineage-determining master control genes whose Wnt-responsiveness is conferred by BCL9, or its paralog B9L (BCL9-Like, or BCL9-2)^8,9^: these factors (called BCL9 below unless a specific paralog is referred to) bind to and capture stabilised β-catenin, to convey it to TCF within the enhanceosome via docking to Pygo, a chromatin-binding PHD finger protein^10^. Wnt/β-catenin signalling specifies numerous cell fate decisions during animal development, and controls self-renewal of virtually every adult tissue, including the intestinal epithelium^11^. This stem cell function could explain why β-catenin is a potent oncogene, as revealed by the prevalence, in many cancers, of activating mutations in β-catenin that render it refractory to degradation^3^.

Despite its considerable oncogenic potential, there are no well-validated inhibitors of activated β-catenin that could be developed as therapeutics, because of the lack of druggable downstream targets^12^. Nevertheless, several small molecules have been reported to inhibit activated β-catenin directly or indirectly, but most of these produce unspecific (off-target) cell toxicity: e.g., tankyrase inhibitors destabilise β-catenin by boosting the levels of Axin^13^, but also of other targets^14^, which may contribute to their toxicity in mice^15^. Furthermore, cells experiencing chronic Wnt signalling such as *APC*-mutant cancer cells proved refractory to tankyrase inhibition, mainly because they express high levels of LEF1 (a TCF paralog) or B9L, which shield β-catenin from Axin-dependent destruction^13^.

The role of BCL9 as an essential co-factor of *Drosophila* β-catenin was demonstrated by genetic studies^16^. In mice, deletion of both *Bcl9* paralogs causes embryonic lethality, while tissue-specific deletion in muscle leads to β-catenin-dependent regeneration defects^17^. Similarly, conditional deletion of both paralogs in the intestine reduces β-catenin-dependent transcription compartment in intestinal crypts, suggesting a role of Bcl9 in specifying ‘stemness’ in this self-renewing compartment^18,19^. BCL9 and B9L are often overexpressed in colorectal cancer cell lines and carcinomas, maintaining their β-catenin-dependent transcription^13,20^, and overexpressed B9L promotes intestinal tumourigenesis^21^.

BCL9 functions as a scaffold of the Wnt enhanceosome^7^. Its binding to the Pygo PHD finger promotes its recognition of methylated histone H3 tail^10,22^. It also binds to the N-terminus of the Armadillo Repeat Domain (ARD) of β-catenin via a short helical domain called HD2^23,24^, an interaction that can be blocked by individual HD2 point mutations^16,25^. Binding of BCL9 to β-catenin can also be blocked by natural compounds^13,23^, stapled helices mimicking HD2^26^ or rationally-designed small molecules^27^, which attenuate β-catenin-dependent transcription in colorectal cancer cells and β-catenin-dependent tumourigenesis in mouse models. These studies have thus provided proof-of-concept for the druggability of BCL9-β-catenin interaction.

In the light of the well-documented role of BCL9 in facilitating β-catenin-dependent transcription, it was puzzling that the conditional double-knockout of *Bcl9* and *B9l* in the intestinal epithelium (*Bcl9/B9l DKO* below) did not reduce the tumour numbers in mouse models based on colitis and chemically-induced β-catenin-dependent tumours^18^, or on weak attenuation of *Apc* function^19^. Here, we re-assess the role of Bcl9 in intestinal tumourigenesis in two mouse models bearing *Apc* mutations, namely *Apc*^*Min*^ which essentially abolishes Apc function, and *Apc*^*1322T*^ which retains partial function of Apc in regulating β-catenin and, importantly, mimics the most prevalent *APC* mutations in human colon cancers^28^. Deletion of *Bcl9* and *Pygo* extends the disease-free life in both models, especially deletion of *Bcl9* which essentially cures *Apc*^*1322T*^ mice of their neoplastic disease, restoring a normal life span in these otherwise moribund mice. RNA profiling reveals that Bcl9 loss synergises with Pygo loss downstream of *Apc* loss to shift the adenomatous gene expression programme from stem cell-like towards differentiation along secretory cell lineages. Our study also uncovers a post-transcriptional effect of Bcl9 deletion in *Apc*^*Min*^ adenoma, namely a striking relocation of their nuclear β-catenin to their cell surface, likely increasing their cell adhesion and retention in crypts, which could account for the numerous tiny adenomas seen in this model. Importantly, this undesirable effect is not observed in *Apc*^*1322T*^ adenomas whose cell surface β-catenin appears normal, likely because the Apc^1322T^ truncation retains binding to, and partial regulation of, β-catenin. Our results from this model therefore illustrate the significant potential of BCL9 and Pygo as targets for therapeutic interference in colorectal cancer.

## Results

### Loss of Bcl9 or Pygo extends the healthy life of *Apc*^*Min*^ mice

**Fig. 1:**
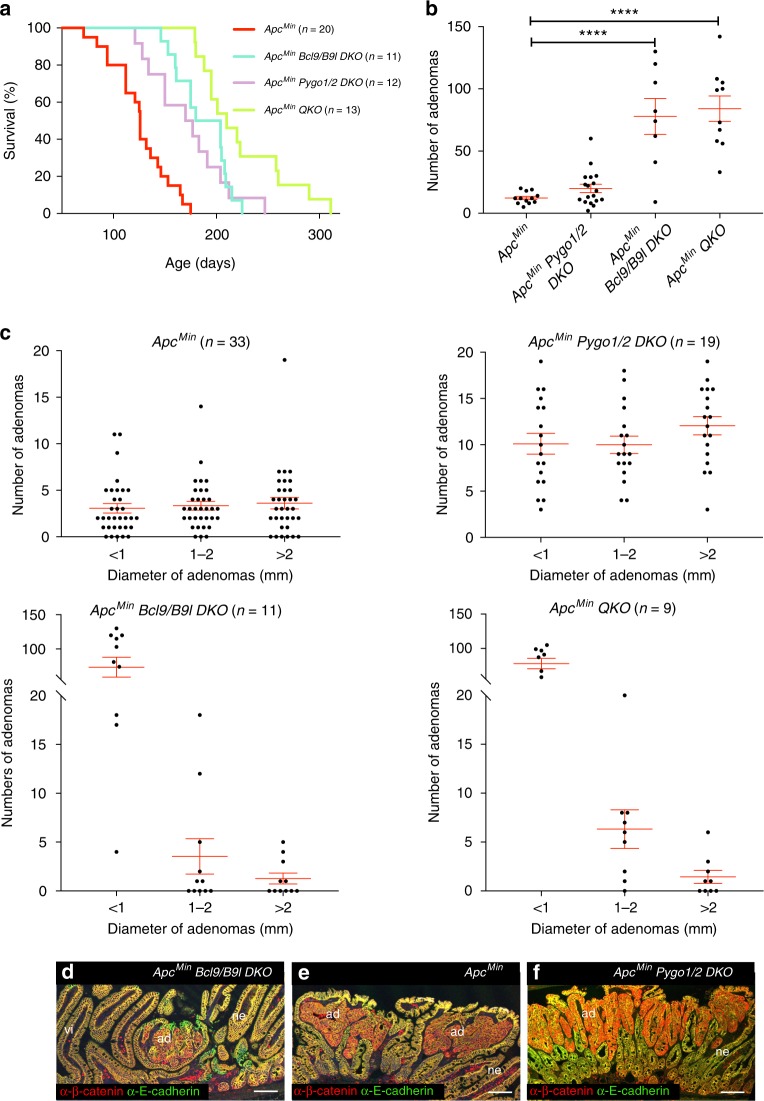
Loss of Bcl9 and Pygo extends the disease-free live of *Apc*^*Min*^ mice. **a** Kaplan–Meier survival plots of *Apc*^*Min*^ *DKO* or *QKO* cohorts and matched *Apc*^*Min*^ controls, as indicated on the right; statistical significance, *p* *<* 0.0001 (log-rank test). **b** Adenoma counts in small intestines from 120 day-old *Apc*^*Min*^ *DKO* or *QKO* and matched *Apc*^*Min*^ control mice, as indicated underneath graph; each dot represents one mouse; mean and SEM indicated by horizontal lines; ****, *p* < 0.0001 (Tukey’s multiple comparisons test). **c** Size distributions of adenomas shown in (**b**); tumours from each mouse (represented by dots) were grouped into three size classes, as indicated underneath graphs; lines as in (**b**). **d**–**f** IF of representative adenomas from *Apc*^*Min*^ *DKO* and matched controls as indicated, after fixation and double-staining with α-β-catenin (*red*) and α-E-cadherin (*green*) antibodies; *QKO* adenomas look similar to *Bcl9/B9l DKO* adenomas (*ad*), of which the majority ( > 95%) do not protrude above the villi (*vi*) and never progress to macroadenoma (unlike *Pygo1/2 DKO* and control adenomas); scale bar, 100 μm. *ne*, normal epithelium. See also Supplementary Figures 1–4

Mice bearing the *Apc*^*Min*^ germline mutation develop some two dozen adenomas in their small intestines, detectable from ~100 days of age, each arising as a result of *Apc* loss-of-heterozygosity (LOH) in an individual intestinal epithelial cell^29^. Half of these mice succumb to their disease by ~120 days (Fig. 1a), turning anemic and losing weight. However, conditional deletion of Pygo or Bcl9 in the intestinal epithelium (with *villin.Cre*)^30^ prolonged the mean disease-free live of these mice by 60–90 days, or even more if all four paralogs were deleted simultaneously (*QKO*; Fig. 1a). The crypt-villus structure appeared normal in these strains, without any detectable changes in the numbers of dividing or apoptotic cells (Supplementary Figure 1, and A.E.K. Ibrahim, unpublished). Clearly, *Apc*^*Min*^ mice benefit from loss of Pygo or Bcl9 in terms of survival, with maximal benefit derived from the simultaneous loss of all four paralogs.

Next, we determined the RNA expression profiles of intestinal crypts from the *DKO* strains using Illumina Bead Arrays, but found only 96 significant (*p* < 0.05) gene expression changes compared to matched controls. Of these, 90% showed the same up- or downregulation trends in the two types of *DKO* (Supplementary Figure 2), as expected since Bcl9 and Pygo act in the same complex^6^. Notably, five histone H2A and four ribosomal protein genes were downregulated in these *DKO* samples, while five Defensin genes were upregulated, including *Defa1* encoding an early differentiation marker for Paneth cells^31,32^. Thus, deletion of Bcl9 or Pygo may promote Paneth cell specification, although there was no detectable increase in the numbers of lysozyme-positive cells (marking fully differentiated Paneth cells)^31,33^ in *DKO* crypt sections (Supplementary Figure 1). Nevertheless, our RNA profiling data suggest that Bcl9 or Pygo loss biases cell fates from proliferative towards differentiated.

### Loss of Bcl9 or Pygo increases tumour numbers in *Apc*^*Min*^ mice

In light of this, it was surprising that the tumour numbers were increased ~3x in small intestines of *Pygo*-deleted *Apc*^*Min*^ mice compared to *Apc*^*Min*^ littermates (Fig. 1b). By contrast, there were far fewer large adenomas (>1 mm) in *Bcl9*-deleted *Apc*^*Min*^ intestines, although these were embedded within a lawn of tiny adenomas (< 1 mm; Fig. 1c). In addition, histological analysis of ‘swiss rolls’ revealed numerous minute adenomas in these intestines buried below the intestinal villi and thus undetectable by surface inspection (Fig. 1d), while the great majority of adenomas in *Pygo*-deleted and *Apc*^*Min*^ control intestines protrude above these villi (Fig. 1e, f). This striking change in size distribution from large to minute adenomas indicates that Bcl9 is required for tumour growth. Note that the increased tumour numbers in *Apc*^*Min*^ *DKO* intestines were unlikely to be caused by elevated LOH, given that the numbers of γ-H2AX positive foci (marking double-stranded DNA breaks and other lesions underlying LOH)^34^ are similarly low as in *Apc*^*Min*^ controls (Supplementary Figure 3). We conclude that loss of Bcl9 in the intestine, despite elevating tumour numbers, slows down the growth of these tumours, suggesting a role of Bcl9 in promoting their proliferation. This is consistent with the findings by Gay et al. (*co-submitted*) that Bcl9 is required for crypt regeneration following irradiation, and for efficient clonal expansion in the murine intestine. Furthermore, B9L also appears to be essential for the proliferation of *APC*-mutant cancer cells: its depletion in SW480 cells (expressing APC truncated at amino acid 1338) attenuated their proliferation substantially, and it proved impossible to recover proliferating cell colonies that lack B9L (Supplementary Figure 4).

The tumour phenotype of the *Apc*^*Min*^ *QKO* intestines was virtually indistinguishable from that of *Bcl9*-deleted *Apc*^*Min*^ intestines (Fig. 1b, c), suggesting that Bcl9 loss may be epistatic over Pygo loss (but see below). Notably, the weight gain of *DKO* and *QKO* mice was normal during their extended life spans, and so their increased tumour loads were well tolerated. Indeed, the morbidity of *Apc*^*Min*^ mice is thought to be caused by their anaemia rather than their adenoma burden^5^.

### Loss of Bcl9 or Pygo shifts cells towards differentiation

**Fig. 2:**
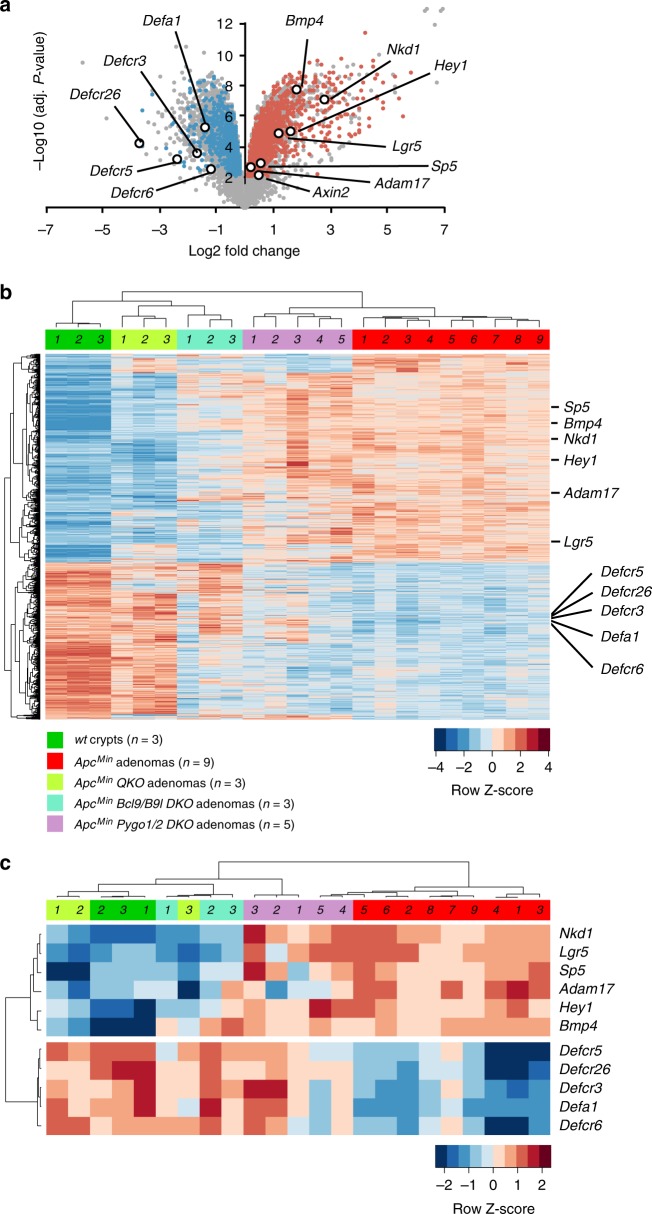
Simultaneous Bcl9 and Pygo loss normalises the *Apc*-mutant gene expression profile of adenomas. **a** Volcano plot showing gene probe changes between *Apc*^*Min*^ adenomas vs normal crypts; *coloured*, set of gene probes showing the strongest trend reversals between *QKO* and control adenomas, as shown in (**b**); labeled are selected Wnt target (*right*) or Defensin genes (*left*), up- (*red*) or downregulated (*blue*) in adenomas vs normal crypts (*p* < 0.01, Benjamini & Hochberg FDR); additional Wnt target genes were identified at a lower significance level (*p* < 0.05, Benjamini & Hochberg FDR) including *Ascl2* (slightly upregulated in adenomas, two independent probes) and *Cdh1* (encoding E-cadherin; slightly downregulated in adenomas, one probe). **b** Heat map showing hierarchical clustering of 1800 gene probe changes (*p* < 0.01, Benjamini & Hochberg FDR) across 5 different cohorts, colour-coded as indicated below; individual samples are designated by numbers in bar; shown are gene probes exhibiting the strongest reversals from adenoma to normal crypts in at least one of the three other cohorts; colours (coded as in **a**) indicate Z-scores of normalised, VST- and batch-adjusted expression values for each individual gene probe. **c** Heat maps as in (**b**) for Wnt target and Defensin genes. See also Supplementary Figures 5 & 6

To test this apparent epistasis between Bcl9 and Pygo, we determined the RNA profiles in adenomas excised from *Apc*^*Min*^ *DKO* and *QKO* small intestines (pooled from 2–3 RNA extractions). Comparing *Apc*^*Min*^ adenomas to normal crypt samples, we found 8852 significant changes (*p* < 0.01), most of which likely represent indirect gene expression changes downstream of master transcription regulators (such as *Ascl2*, *Math1* or *c-myc*; see below). They include upregulation of known Wnt targets (such as *Lgr5*), H2A and ribosomal protein genes, and downregulation of Defensin genes, e.g., *Defa1* (Fig. 2a and Supplementary Figure 2). Consistent with this, acute inactivation of *Apc* in the intestinal epithelium causes crypt expansions, and cell-fate shifts from differentiated to proliferative^35^. Hierarchical clustering based on Euclidean distance confirmed that profiles within samples of the same genotypic cohort were more similar to one another than to those from different cohorts (Fig. 2b, *top*).

Comparing *Apc*^*Min*^ to *QKO* adenomas, we found 2806 significant changes of which 62.3% were dependent on *Apc*, i.e., the corresponding gene probes showed differential expression in adenomas versus (vs) normal crypts; of these, 82.9% showed opposite trends in *QKO* vs *Apc*^*Min*^ controls (Supplementary Figure 5). Furthermore, a substantial fraction of the *QKO*-dependent changes were trend reversals towards the RNA profile of normal crypts (Fig. 2b), including most Wnt target genes (Fig. 2c) except for *Axin2*, a gene constituting a negative feedback loop with elevated expression in *Apc*-mutant adenomas^36^ which was unaffected by loss of Pygo and Bcl9. Indeed, these ‘normalisations’ also extended to previously identified stem cell signatures downstream of Wnt/TCF^37^, EphB2 or Lgr5^38,39^ (Supplementary Figure 6), consistent with previous results^18,19^. Thus, simultaneous deletion of Bcl9 and Pygo shifts the transcription programme of *Apc*^*Min*^ adenomas from stem cell-like towards normal crypts.

### Synergy between loss of Bcl9 and Pygo in normalising tumours

Our RNA profiling data suggested that Bcl9 loss contributed rather more than Pygo loss to the *QKO*-dependent trend reversals, perhaps explaining why the tumour phenotypes of the *QKO* mice resembled those of *Bcl9/B9l* rather than *Pygo1/2 DKO*. To test this, and to address whether Bcl9 is epistatic over Pygo at the level of transcription, we conducted systematic Pearson’s correlations of the significant changes between *QKO* and control adenomas across all five cohorts.

**Fig. 3:**
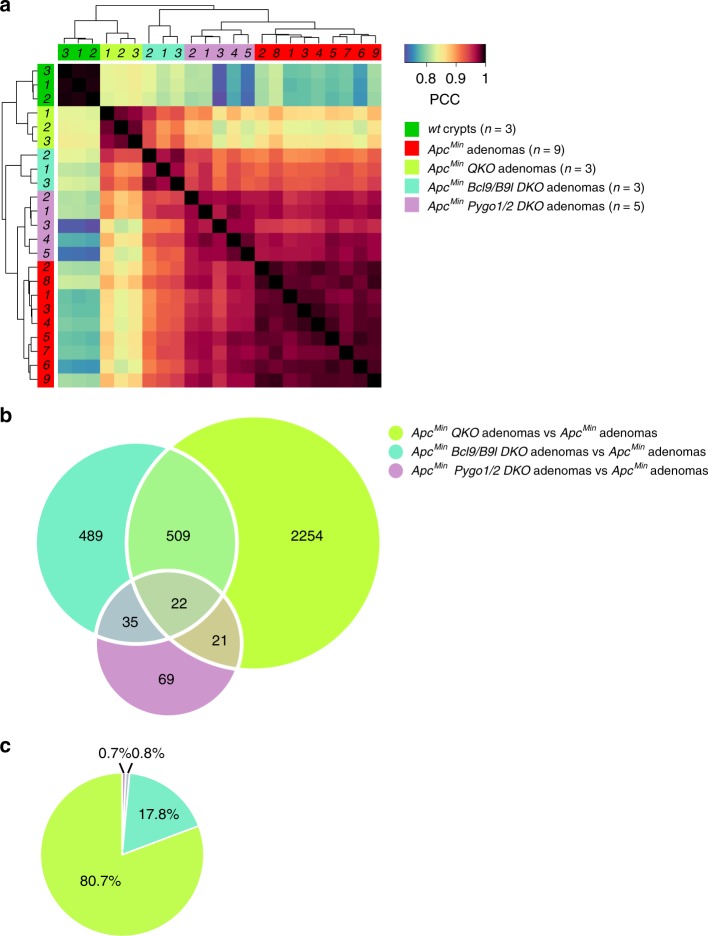
The gene expression profile of *QKO* adenomas is determined largely by Bcl9 loss. **a** Pearson correlation heat-map of gene probe changes of *QKO* vs control adenomas across all 5 cohorts (see key, for genotypes); PCC, Pearson correlation coefficient (*red*, high; *blue*, low). **b** Venn diagram showing number of gene probe changes in cohorts (individual and shared), as shown in key. **c** Pie chart showing the unique contribution of genotypes shown in (**b**) to gene probe changes of *QKO* vs control adenomas. See also Supplementary Figure 7

While these correlations revealed some resemblance between the two types of *DKO* samples, the *QKO* samples more closely resembled the *Bcl9/B9l DKO* than the *Pygo1/2 DKO* (Fig. 3a): *QKO* and *Bcl9/B9l DKO* cohorts shared 531 changes (< 15.6% of the Bcl9-regulated genes), *QKO* and *Pygo1/2 DKO* cohorts shared 43 changes (< 1.3% of the Pygo-regulated genes; Fig. 3b), and 22 changes were shared between all three cohorts (Supplementary Figure 7). Importantly, the vast majority of the *QKO*-dependent changes (80.7%) were unique and not shared with either *DKO* cohort (Fig. 3c).

Two conclusions can be drawn: firstly, a large fraction of the Bcl9- and Pygo-regulated genes are downstream of *Apc*, consistent with Bcl9 and Pygo acting predominantly on *Apc*-regulated genes in these tumours. Secondly, although more *Apc*-regulated genes are affected by Bcl9 rather than Pygo loss, simultaneous deletion of both paralog pairs affects vastly more *Apc*-regulated genes than deletion of either alone. An important corollary is that Bcl9 loss strongly synergises with Pygo loss in shifting the adenoma gene expression programme towards that of normal crypts, and argues against an epistatic relationship between the two enhanceosome factors.

### Loss of Bcl9 or Pygo promotes differentiation towards secretory cell fates

**Fig. 4:**
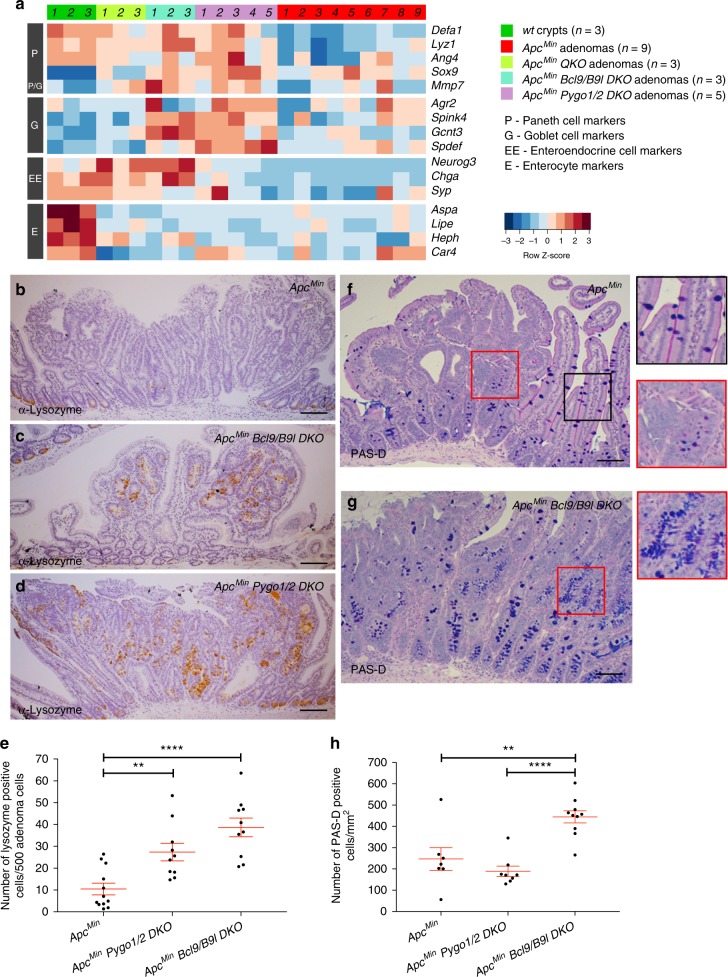
Loss of Bcl9 or Pygo causes a shift towards secretory cell fates in adenomas. **a** Heat map (as in Fig. 2b) for selected differentiation markers, as indicated on the right. **b**–**g** Sections through adenomas (genotypes indicated in panels) stained for (**b**–**d**) Paneth (*brown*, α-lysozyme) or (**f**, **g**) goblet cells (*dark blue*, PAS-D); *right*, magnified views of squares in (**f**, **g**), with strong PAS-D signals in villi (*black square*) or adenomas (*red squares*) indicating mature or immature goblet cells, respectively; *purple*, hematoxylin; scale bar, 100 μm. **e**, **h** Quantification of (**b**–**g**)

*Defa1* was our top hit amongst the changes shared between all three *DKO* and *QKO* cohorts (Supplementary Figure 7). Indeed, lysozyme and other Paneth cell markers behaved similarly, showing a tendency to be upregulated in both types of *DKO* vs control adenomas (Fig. 4a). Immunohistochemistry (IHC) revealed 2 × 3 more lysozyme-positive cells in *DKO* adenoma vs *Apc*^*Min*^ control sections (Fig. 4b–e), confirming that the former contain more Paneth cells than adenomas with functional Bcl9 or Pygo.

We also found upregulation trends of markers for the other two cell types of the secretory lineage, enteroendocrine and goblet cells^31^, in *DKO* vs control adenomas (Fig. 4a). We confirmed this for goblet cells, using Period-Acid Schiff (PAS) diastase (PAS-D) staining^33^: we found about twice as many PAS-D-positive cells in *Bcl9/B9l DKO* vs control adenomas (Fig. 4f–h), although the adenomatous PAS-D signals (labelling intracellular mucus) were smaller than those from normal goblet cells (Fig. 4f, g; high-magnification views), suggesting that these metaplastic goblet cells are not fully differentiated. Thus, loss of Pygo or Bcl9 promotes secretory cell metaplasia, apparently at the expense of stem cell-like fates, given the downregulation of Lgr5 and stem cell signature genes in these adenomas (Supplementary Figure 6). In contrast, markers for absorptive enterocytes (constituting the bulk of intestinal villi) were not significantly changed (Fig. 4a). This is consistent with the notion that enterocytes represent the ‘default’ cell fate within the intestinal epithelium^31^ whose specification requires neither β-catenin nor *Math1*^32,33^.

### Redistribution of β-catenin in *Bcl9*-deleted *Apc*^*Min*^ adenomas

The above-described RNA profile changes do not fully explain why the tumour phenotypes caused by Bcl9 loss differed from those in *Pygo*-deleted *Apc*^*Min*^ intestines. We thus surmised that the underlying cause may be post-transcriptional. One key determinant for tumour multiplicity is the β-catenin-dependent cell adhesion between tumour and adjacent normal cells which, if elevated, decreases the rate of tumour loss through the normal epithelial renewal process^40,41^ through upwards displacement along the crypt-villus axis^31^, thereby increasing tumour numbers. Notably, APC regulates the junctional pool of β-catenin^42^, and complete Apc loss-of-function causes a drastic increase of nucleocytoplasmic at the expense of junctional β-catenin^35^. We thus examined the subcellular distribution of β-catenin in *Bcl9*-deleted *Apc*^*Min*^ adenomas by immunofluorescence (IF).

**Fig. 5:**
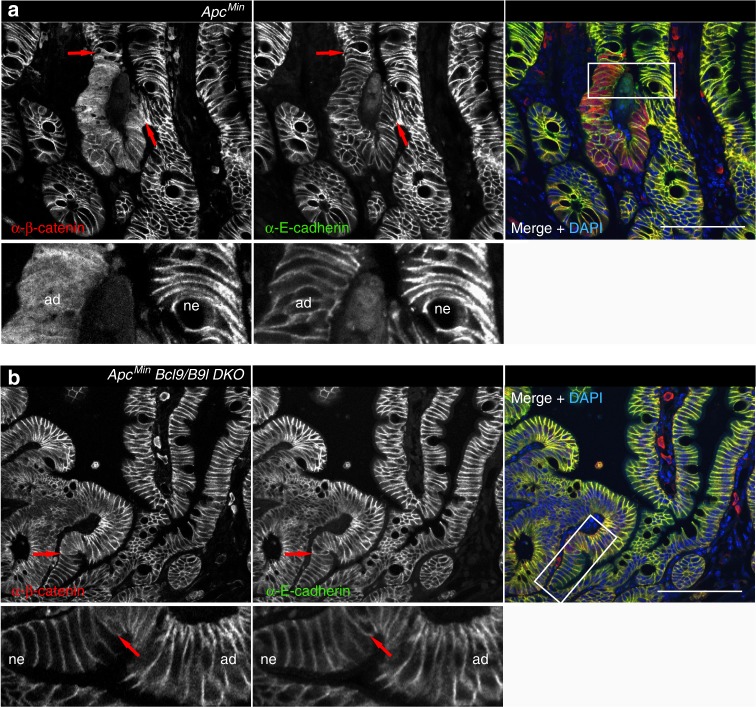
Loss of Bcl9 causes relocation of β-catenin to the cell surface of *Apc*^*Min*^ adenoma cells. IF of nascent adenomas from **a** *Apc*^*Min*^ or **b** *Bcl9/B9l DKO* small intestines, after fixation and double-staining as in Fig. 1d–f; *arrows*, boundaries between normal epithelium (*ne*) and adenoma (*ad*); underneath, magnified views of squares (in merges, *right*) containing comparable normal and adenomatous epithelial cells; *blue*, nuclei marked by DAPI (only in merges); scale bars, 50 μm. Note the high levels of nucleocytoplasmic β-catenin in *Apc*^*Min*^ adenomas (strong red fluorescence in (**a**); see also Fig. 1e, f), relocated to the cell surface in *Bcl9/B9l DKO* adenomas (yellow fluorescence in **b**), leaving barely any discontinuities between adenomatous and adjacent normal epithelial cells

As expected, *Apc*^*Min*^ adenoma cells exhibited high levels of cytoplasmic and nuclear β-catenin (Fig. 5a, red signal), but low levels of junctional β-catenin compared to adjacent normal epithelial cells. In contrast, *Bcl9/B9l DKO* adenoma cells showed very little nucleocytoplasmic β-catenin, which explains the low levels of Wnt target gene expression in these *Bcl9*-deleted adenomas. Instead, we observed prominent β-catenin staining at the cell surface of these tumours, coinciding with strong E-cadherin staining (Fig. 5b, yellow). Indeed, in these sections, the membraneous β-catenin staining patterns barely differed between adenomas (which showed slightly elevated nucleocytoplasmic β-catenin, allowing their identification) and adjacent normal epithelium (Fig. 5b, insets). In contrast, the β-catenin staining patterns in *Pygo*-deleted adenomas looked similar to those in *Apc*^*Min*^ controls (Fig. 5a, insets), and so even the tiniest nascent tumours stand out by their strong nucleocytoplasmic β-catenin signal^43^. The total levels of β-catenin and E-cadherin are comparable in *DKO* and *Apc*^*Min*^ control adenomas (Supplementary Figure 8). It thus appears that, in the absence of its nuclear binding partner Bcl9, β-catenin cannot be retained in the nucleus and is thus sequestered at the cell surface by its alternative binding partner E-cadherin (see Discussion).

### Loss of Bcl9 cures *Apc*^*1322T*^ mice of their neoplastic disease

**Fig. 6:**
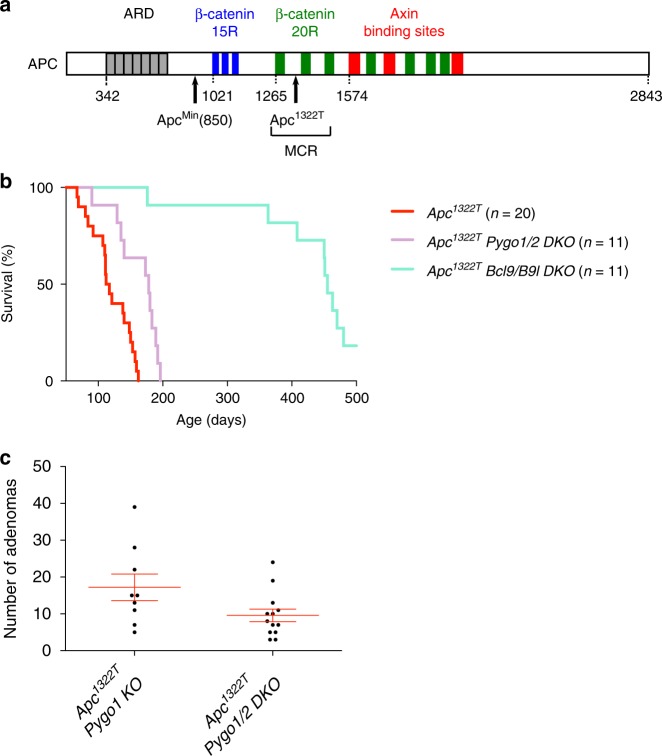
Loss of Bcl9 suppresses the neoplastic disease of *Apc*^*1332T*^ mice. **a** Cartoon of human APC, with Axin (*red*) and β-catenin binding sites (15R1-3, *blue*; 20R1-7, *green*) in its central portion downstream of the ARD (*grey*); *numbers*, N-terminal limits of motifs; *arrows*, truncation endpoints of *Apc*^*Min*^ and *Apc*^*1332T*^ alleles; *bracket*, mutation cluster region (MCR) in human colorectal cancers (see text). **b** Kaplan–Meier survival plots of *Apc*^*1332T*^ cohorts, as in Fig. 1a; statistical significance, *p* < 0.0001 (log-rank test). **c** Adenoma counts in small intestines from 77 day-old *Apc*^*1332T*^ *Pygo1/2 DKO* and matched *Apc*^*1332T*^ *Pygo1 KO* controls, as in Fig. 1b; statistical significance, *p* < 0.05 (*t-*test). See also Supplementary Figures 9 & 10

While our analysis was in progress, a new model for colorectal cancer became available^28^, based on mice bearing an *Apc* allele (*Apc*^*1322T*^) that mimics one of the most commonly observed *APC* mutations observed in human carcinomas. The *Apc*^*1322T*^ truncation is substantially longer than the Apc^Min^ truncation and, unlike the latter, retains multiple β-catenin binding sites (Fig. 6a) and, thus, partial function in downregulating β-catenin. Notably, a wealth of genetic data from human colorectal cancers indicates a strong positive selection for the retention of β-catenin binding sites^44^, i.e., for the preservation of partial APC function (‘just-right signalling’)^45^. Indeed, colorectal carcinomas that express Apc^Min^-like truncations without any β-catenin binding sites are exceedingly rare^44^.

We thus repeated our analyses in the *Apc*^*1322T*^ model. RNAseq analysis of adenomas from 100–120 day old *Apc*^*Min*^ and *Apc*^*1322T*^ mice revealed only 178 significant changes, a minute fraction of the > 8000 significant changes caused by the *Apc*^*Min*^ mutation itself. We thus expect the above-described RNA profiling data from *Apc*^*Min*^ adenomas to pertain also to *Apc*^*1322T*^ adenomas, at least qualitatively. However, Wnt target genes tended to be less upregulated in *Apc*^*1322T*^ compared to *Apc*^Min^ adenomas (Supplementary Figure 9), consistent with the Apc^1322T^ truncation retaining partial function in downregulating β-catenin.

Despite this, *Apc*^*1322T*^ mice develop more rapid and severe polyposis, and succumb to their neoplastic disease earlier, than *Apc*^*Min*^ mice^28^. Under our conditions, *Apc*^*1322T*^ mice typically showed overt disease by ~110 days, but their disease-free life was extended to ~180 days upon Pygo deletion (Fig. 6b). Furthermore, the mean tumour numbers in *Pygo*-deleted *Apc*^*1322T*^ intestines were < 10, compared to ~18 in control littermates (Fig. 6c) by day 77 (i.e. scored 43 days earlier than those of *Apc*^*Min*^ mice). The size distribution of these tumours was the same in the two cohorts (Supplementary Figure 10). Thus, the loss of Pygo alleviated the neoplastic disease in this model considerably.

Strikingly, the *Bcl9*-deleted *Apc*^*1322T*^ intestines showed no adenomas whatsoever at 77 days, and about half of these mice remained disease-free beyond ~450 days (Fig. 6b). Indeed, two mice were still healthy at our experimental end-point (day 500). Examining each of the 9 mice aged > 400 days, we found < 11 adenomas per intestine, which probably developed after ~200 days since younger mice (2 aged 77, and 2 aged 200 days) showed only one adenoma between them (at 200 days), while the remaining 3 intestines showed none. Intestinal swiss rolls of 3 mice aged > 400 days confirmed that these were completely normal, without any adenomas. Thus, Bcl9 loss essentially cures *Apc*^*1322T*^ mice of their neoplastic disease.

### *Apc*^*1322T*^ adenomas show normal cell surface β-catenin

**Fig. 7:**
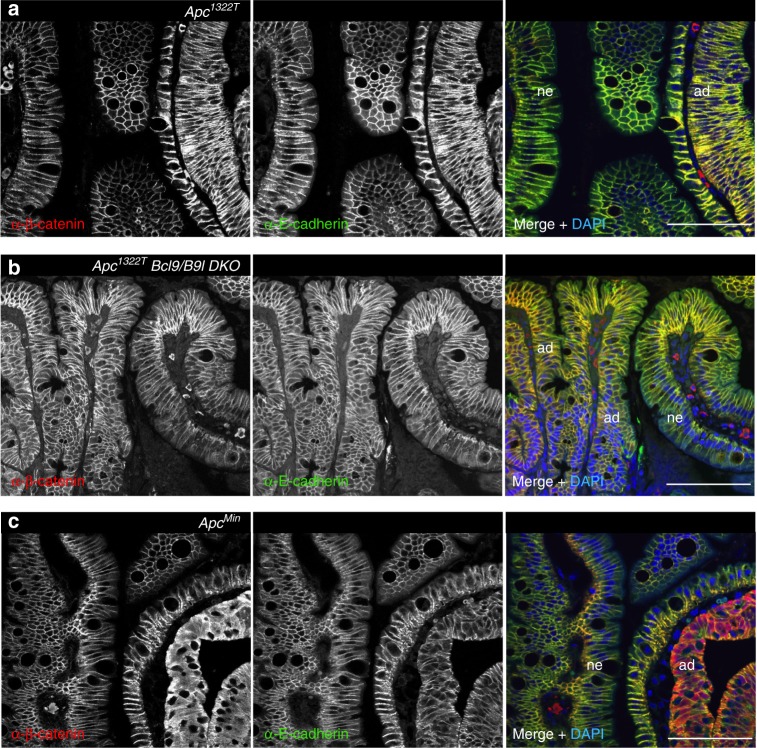
The subcellular distribution of β-catenin in *Apc*^*1332T*^ adenoma cells is not sensitive to Bcl9 loss. IF of adenoma (*ad*) from (**a**) *Apc*^*1332T*^, (**b**) *Apc*^*1332T*^ *Bcl9/B9l DKO* or *Apc*^*Min*^ small intestines, and adjacent normal epithelia (*ne*), fixed and stained as in Fig. 5; scale bars, 50 μm. The nucleocytoplasmic β-catenin levels are barely elevated in *Apc*^*1332T*^ adenomas (compared to *Apc*^*Min*^ adenoma, red fluorescence in **c**) and their cell surface β-catenin is high (yellow fluorescence in **a**, **b**) regardless of Bcl9

Given the similar RNA profiles between the two models, we surmised that the root cause for their different responses to Bcl9 loss might be post-transcriptional. Indeed, we detected only a subtle increase in nucleocytoplasmic β-catenin staining in *Apc*^*1322T*^ adenomas, barely delineating these tumours (Fig. 7a), consistent with previous results^28^ and with the notion that the Apc^1322T^ truncation retains moderate function in downregulating β-catenin. As might be expected, there was no detectable discontinuity in cell surface β-catenin between adenomatous and adjacent normal epithelial cells in *Apc*^*1322T*^ *Bcl9/B9l DKO* intestines (Fig. 7b), in stark contrast to the overt discontinuities in *Apc*^*Min*^ adenomas (Fig. 7c).

## Discussion

We used two mouse tumour models based on different *Apc* germline mutations (*Apc*^*Min*^ and *Apc*^*1322T*^) to assess the roles of the Wnt enhanceosome components Bcl9 and Pygo in intestinal neoplasia. In both models, deletion of these factors suppressed tumourigenesis, and extended the disease-free life spans, possibly owing to a shift in the gene expression of *Apc*-mutant adenomas from stem cell-like towards differentiated. Importantly, simultaneous deletion of Bcl9 and Pygo proved vastly more effective in reversing the neoplastic transcription programme towards normal than deleting either of them alone. Our results underscore the notion that these factors synergise to facilitate the docking of β-catenin to the Wnt enhanceosome^6,7^, to co-activate transcription of *Apc* target genes. In addition, we uncovered evidence for increased tumour retention in *Bcl9*-deleted *Apc*^*Min*^ intestines, but this undesirable effect was not evident in the *Apc*^*1322T*^ model. Indeed, intestinal deletion of Bcl9 blocked tumourigenesis in these mice and essentially cured them of their neoplastic disease. Since this model recapitulates common human colorectal carcinomas in terms of their *APC* mutations^28^, this indicates the potential of BCL9 as a therapeutic target in this often lethal cancer.

Several of our results support the Wnt enhanceosome model and, indeed, can be explained by it even though this model, derived from biochemical and functional data from *Drosophila* and human cells (including *APC*-mutant colorectal cancer cells) has not been validated specifically for murine cells. For example, we observed a strong synergy between Bcl9 loss and Pygo loss in the suppression of neoplastic disease (Fig. 1a), and in the shift of adenomatous gene expression from neoplastic towards normal (Figs 2b and 3a), consistent with this model. However, we also uncovered clear quantitative differences between the two conditions in that the effects of Bcl9 loss on gene expression and tumour phenotypes were consistently stronger than those of Pygo loss, indicating that Bcl9 is more important than Pygo in enabling β-catenin to co-activate TCF-dependent transcription. This is consistent with its scaffold function within the Wnt enhanceosome^7^, whereby Bcl9 contacts Pygo as well as the Chip/LDB-SSDP (ChiLS) core complex of the enhanceosome and so only partially relies on Pygo for its association with the enhanceosome. Also, the Wnt enhanceosome may retain partial integrity and function in the absence of Pygo owing to its linkage to the BAF chromatin remodelling complex^46^ whose two PHD fingers might substitute for Pygo’s PHD finger in conferring chromatin association. Crucially, Bcl9 confers Wnt responsiveness on this multiprotein complex as its only component that can bind to and recruit β-catenin. This, together with the scaffolding role of Bcl9, readily explains why the transcriptional profile of the *QKO* tumours is largely determined by Bcl9 loss (Fig. 3b, c) and, by implication, why the latter has such a profound impact on β-catenin-dependent intestinal neoplasia in both *Apc* models (Figs 1a and 6b).

We also observed qualitative differences between Bcl9 and Pygo loss regarding the tumour phenotypes. The most striking one was a marked shift in adenoma size from large to tiny in *Bcl9*-deleted *Apc*^*Min*^ mice, while Pygo loss had no effect on the tumour size distribution, implying a role of Bcl9 but not Pygo in promoting tumour growth. However, this apparent qualitative difference might simply reflect a threshold effect: in the absence of Bcl9, expression of master regulators of tumour growth such as *c-myc*^47^ might be reduced sub-critically, which could translate into an off-switch of downstream effector programmes, while the effect of Pygo loss on these genes might be too weak to toggle this switch. This would explain why far fewer genes were differentially expressed in *Pygo*-deleted compared to *Bcl9*-deleted tumours. It is also conceivable that some *Apc* target genes are controlled only by Bcl9 but not Pygo, but the synergy of their simultaneous deletion argues against this. Also, the qualitative differences in tumour phenotypes between the two *DKO* strains were only apparent in the *Apc*^*Min*^ model (for reasons discussed below) while the *Apc*^*1332T*^ mice responded to Bcl9 loss similarly albeit more profoundly than to Pygo loss.

Many of the Wnt enhanceosome components including its ChiLS core and Groucho/TLE co-repressor were discovered as Notch signalling effectors in *Drosophila*^48^. ChiLS is recruited to target enhancers via direct binding to members of the basic helix-loop-helix (bHLH) proteins, such as the mammalian Notch effectors Ascl (Achaete/scute-like) and Math/Atoh (Atonal homolog); these positively-acting enhancer-binding proteins compete with negatively-acting Hairy/enhancer-of-split (Hes) factors, Notch effectors whose expression is universally induced by Notch signalling, and which silence downstream enhancers by recruiting Groucho/TLE^49^. We thus proposed that the Wnt enhanceosome integrates Wnt and Notch signalling inputs^6^, relying on Bcl9-Pygo and positively-acting Notch effectors for its activation, and on Notch-dependent Hes repressors for its re-repression.

This notion is reinforced by our finding that loss of Bcl9 or Pygo tends to shift cell fates from stem-cell like towards differentiation along all three secretory cell lineages of the intestinal epithelium. Each of these cell types requires opposing inputs from Wnt and Notch signalling^50^, and their fates appear to be specified stochastically in transit-amplifying cells located above the crypt base, descending from Lgr5-positive crypt cells^51^. Their specifications depend on β-catenin^33^ and the master regulator Math1^32^, while their differentiation along specific secretory lineages requires repressive inputs from Hes1 and other redundant Hes factors^52^ whose localised accumulation depends on Notch signalling from neighbouring cells^31^. If *Math1* were controlled by the Wnt enhanceosome, a molecular device for integrating opposing inputs from β-catenin and Notch > Hes signalling, this would provide a mechanistic explanation for the results by Tian et al.^50^.

Paneth cells serve as stem cell niches that provide Wnt and Notch signals to reinforce Lgr5-positivity of adjacent cells^53^. This seemed at odds with the observed reductions of Lgr5 itself, and its downstream signatures, in *Pygo*- or *Bcl9*-deleted adenomas^18^ (Supplementary Figure 6) despite their Paneth cell metaplasia. However, these metaplastic Paneth cells may only be partially differentiated (Fig. 4a) and, thus, not fully functional stem cell niches. Other differentiation markers also failed to be upregulated in metaplastic secretory cells (Fig. 4a), e.g., metaplastic goblet cells seemed partially differentiated judging by their petite PAS-D signals in *DKO* adenomas (Fig. 4f, g). However, the differentiation of enterocytes appeared unaffected (Fig. 4a), as expected since these absorptive cells constitute the ‘default’ fate adopted by intestinal epithelial cells in the absence of β-catenin and Math1^31–33^.

Our results thus underscore the notion that the Wnt enhanceosome operates in cellular contexts, and on target genes encoding cell lineage-determinants whose regulation requires integration of Wnt and Notch signalling, such as *Math1* (whose expression was under our detection threshold; Fig. 2a). Another candidate for an enhanceosome-controlled gene is *Ascl2*, a lineage-determining gene for crypt stem cell fates^54^. Finally, c-myc responds to Notch via a remote enhancer^55^ and to TCF/β-catenin via proximal *cis*-regulatory sequences^56^, which may be integrated by the Wnt enhanceosome whose ChiLS core confers communication between promoters and remote enhancers^6^. Note that upregulation of *c-myc* by Wnt/TCF is subtle^56^ albeit functionally relevant^47^ which explains why this gene did not feature in our lists of significant expression changes (Fig. 2 and Supplementary Figure 5).

Our comparative analysis of the two *Apc* models revealed an unexpected post-transcriptional effect of Bcl9 loss on β-catenin. Tumourigenesis in each of these models is initiated by an Apc truncation of which the longer (Apc^1332T^) retains binding to β-catenin and, thus, partial function regarding β-catenin’s roles in transcription and adhesion^57^ whereas the shorter Apc^Min^ truncation does not, and so *Apc*^*Min*^ is equivalent to a null allele with regard to β-catenin regulation. The tumours produced by two models are morphologically indistinguishable, and exhibit very similar RNA profiles^28,58^, although the Wnt and stem cell signature genes tended to be more upregulated in *Apc*^*Min*^ than in *Apc*^*1332T*^ adenomas (Supplementary Figure 9). This is consistent with results from the human colorectal cancer cell line COLO320 whose APC truncation is Apc^Min^-like and atypically short for human cancers^59^ and which exhibit 2–3 fold higher levels of β-catenin-dependent transcription compared to typical colorectal cancer cells^60^ whose longer APC truncations retain moderate function in downregulating β-catenin. The low levels of junctional β-catenin in *Apc*^*Min*^ tumours may be explained if their dysfunctional *Apc*^*Min*^ truncation were unable to maintain junctional β-catenin^42^. By contrast, the partially functional Apc^1332T^ truncation may maintain near-normal junctional β-catenin in adenomas (Fig. 7a) which could boost their adhesion to neighbouring cells, thereby enhancing their retention within intestinal crypts^41^, and resisting their upward displacement to the villar apices and shedding into the gut lumen. It would explain why *Apc*^*1332T*^ mice develop more severe, and faster, intestinal polyposis than *Apc*^*Min*^ mice^28^, despite their lower Wnt and stem cell signatures.

The same could also explain why the *Bcl9*-deleted *Apc*^*Min*^ intestines exhibited hundreds of tiny adenomas (Fig. 1): evidently, their growth was stunted, owing to their dysfunctional β-catenin-dependent transcription (Fig. 2 and Supplementary Figure 6), but they appeared to be retained more efficiently within intestinal crypts owing to their high levels of cell surface β-catenin (Fig. 6b). Our evidence suggests that the latter reflects sequestration of β-catenin at the cell surface by its alternative binding partner E-cadherin, following its leakage from the nucleus in the absence of Bcl9: it is well established that β-catenin shuttles freely between nucleus and cell surface, regardless of its phosphorylation status, and that its partitioning between these subcellular compartments is determined by retention via its binding partners in these locations^40,61–63^. This redistribution of β-catenin from the nucleus to the cell surface owing to Bcl9 loss was not detectable in *Pygo*-deleted *Apc*^*Min*^ adenomas, likely because β-catenin remains associated with nuclear Bcl9 in this case. We note that loss of E-cadherin alone does not necessarily result in activation of β-catenin-dependent transcription^64^ although the levels of E-cadherin can certainly modulate the nuclear activity of β-catenin in some cellular contexts, e.g., impacting on tumourigenesis in the murine colon^40^.

Therefore, this undesirable effect of Bcl9 deletion on tumour retention in the *Apc*^*Min*^ model partially negates the beneficial effects of Bcl9 loss in curbing β-catenin-dependent neoplastic gene expression. Importantly, there was no detectable relocation of β-catenin in *Apc*^*1332T*^ adenomas, likely because the Apc^1332T^ truncation retains partial activity in downregulating transcriptionally active β-catenin, keeping its nucleocytoplasmic levels relatively low and its cell surface levels normal. This truncation therefore buffers the negative effects of nuclear β-catenin efflux in the absence of Bcl9. The same is expected in the great majority of colorectal carcinomas whose APC truncations, like Apc^1332T^, retain binding to β-catenin and regulating its activity.

The *Apc*^*1332T*^ model has allowed us to discover a crucial role of Bcl9 and Pygo in effecting intestinal neoplasia in a model with high relevance for colorectal cancer. Similar conclusions were reached by Gay et al. (*co-submitted*) who also found Bcl9 to be critical for tumourigenesis in different β-catenin-dependent murine models. Furthermore, our results are broadly consistent with evidence that excess Bcl9-2 or Pygo2 promote tumour progression in the intestine^21,65,66^ and in other tissues^67,68^. Our study also revealed that Bcl9 is more critical for intestinal neoplasia than Pygo, although both proteins synergise to effect β-catenin-dependent transcription. Thus, BCL9 and B9L have significant potential as targets for therapeutic intervention in colorectal cancer. Since proof-of-concept has been obtained for the discovery of reagents that block their binding to β-catenin^23,26,27^, it might be possible to identify inhibitors of this interaction that can be developed into therapeutics.

## Methods

### Animal procedures

Animal care and procedures were performed in accordance with the standards set by the United Kingdom Home Office. For tests in the *Apc*^*Min*^*/*+ model, *Bcl9*^*LoxP/LoxP*^, *B9l*^*LoxP/LoxP*^, *villin*.*Cre* or *Pygo1*^−/−^, *Pygo2*^*LoxP/LoxP*^, *villin.Cre* were back-crossed into a C57BL/6 background for 4 successive generations^5^ whereby the final cross was with *Apc*^*Min*^/+. To generate compound *DKO Apc*^*Min*^ or *Apc*^*1322T*^ strains, *Apc*^*Min*^/+ or *Apc*^*1322T*^*/*+ males were crossed into a homozygous background of *Bcl9*^*LoxP/LoxP*^, *B9l*^*LoxP/LoxP*^ or *Pygo1*^−/−^, *Pygo2*^*LoxP/LoxP*^ also bearing *villin.Cre*/+. Similarly, for the *QKO Apc*^*Min*^/+ strain, *Apc*^*Min*^/+ males were crossed into a homozygous background of *Bcl9*^*LoxP/LoxP*^, *B9l*^*LoxP/LoxP*^, *Pygo1*^−/−^, *Pygo2*^*LoxP/LoxP*^ also bearing *villin.Cre*/+. Only males bearing *Apc* alleles were used for breeding.

For genotyping, tissue from ear biopsies was used. DNA was extracted using TaqMan Sample-to-SNP Kit (ThermoFisher Scientific). For genotyping of *Apc*^*Min*^, the Custom TaqMan SNP Genotyping Assay with the primer set AHX1JET and GTXpress Taqman was used. All other alleles were genotyped by standard PCR with KOD DNA polymerase (Merck Millipore), using the following primers:

*Apc*^*1322T*^: (F) 5’-GATGTAACTCGGTCAGCTGAAGAT-3’; (R-1322T) 5’-GCTTGACGTCACCGGTTCTAG-3’; (R-WT) 5’-TGACAGAAGTACACCTGCTGAATACGA-3’

*Villin.Cre*: (F) 5’-CCGGGTGGGCAGGGTAGAGG-3’; (R) 5’-CATCACTCGTTGCATCGACC-3’

*Bcl9*: (F) 5’-CCACCAAGGAATCGCAGACGT G-3’; (R-LOX) 5’-TGCAACTGAGCTGGGATGTTTGC-3’; (R-WT) 5’-GCTGGGCCCATGCTTGCTC-3’

*B9l*: (F) 5’-TGTCCTCCCTACCTTCCCCTTGG-3’; (R-LOX) 5’-GCGAGGTTAACGTCCCCCAAATC-3’; (R-WT) 5’-TTGCTCAAGATGGCCAGGATGC-3’

*Pygo1*: (F) 5’-ATGGTCCGCATATATTTCTG-3’; (R-LOX) 5’-TGGCGCCCAGCACATAGAC-3’; (R-WT) 5’-CCCCCCATTAATCTCATTTC-3’

*Pygo2*: (F) 5’-AGGGGGATGAGGACAGTG-3’; (R) 5’-GGCGAACTCCGTCAGATG-3’

*Apc*^*Min*^ and *Apc*^*1322T*^ mice were monitored for signs of neoplastic disease; their end point was upon presentation of two signs of the disease (anaemia, weight loss, or reduced mobility). Tumours were scored in dissected small intestines at day 77 (*Apc*^*1322T*^) or 120 (*Apc*^*Min*^). To label proliferating cells, mice were injected intraperitoneally with 150 μl of Bromo-deoxyuridine (BrdU) at 10 mg ml^−1^ in saline, and small intestines were dissected 2 h post injection.

### Intestinal tissue preparations

Intestines were flushed with phosphate-buffered saline (PBS) and cut open lengthwise, to remove any remaining mucosa with Kimcare tissue (Kimberly-Clark, Irving TX, USA). For tumour scoring, the colons were removed at the cecum, and the remaining small intestines were fixed in methacarn solution (60% absolute methanol, 30% chloroform, 10% glacial acetic acid, each v/v) for 24 h. Fixed intestines were inspected under a stereo dissecting microscope, for classification of adenomas into macro- and miniadenomas (see Results).

For protein and RNA extraction, individual adenomas were excised, flash-frozen in liquid nitrogen, and stored at −80 °C for <24 months. To isolate intestinal crypts, small intestines were cut into 5 mm fragments and washed 10x with ice-cold PBS. Fragments were then incubated in 25 ml PBS containing 2 mM EDTA at 4 °C for 30 min, washed with ice-cold PBS, and transferred into 10 ml PBS. Epithelial cells were extracted through vigorous pipetting. The extraction step was repeated 4x with fresh PBS to collect 4 fractions, after which the fractions were pelleted at 300 × *g* at 4 °C for 5 min. Fractions 2–4 were pooled and stored at −80 °C for <24 months.

For western blots, intestinal epithelia were separated from their underlying mucosa by scraping small intestines with a blade, flash-frozen in liquid nitrogen and stored at −80 °C for <24 months.

For IF and IHC, small intestines were fixed in formalin for 24 h and stored in 80% ethanol for <7 days. Swiss rolls were dehydrated by the following successive incubations: 1x in 70% ethanol for 45 min; 1x in 90% ethanol for 1 h; 4x in 100% ethanol for 75 min; 3x in xylene for 90 min. Subsequently, they were embedded in paraffin by incubating 3x in Tek Wax (SAKURA, no. 4659) for 90 min at 60 °C. Sections were cut at 3.5 μm with a Leica microtome RM255.

### IF and IHC

For IHC, slides were de-paraffinized and rehydrated by overnight incubation at 65 °C and subsequently washed as follows: 2 × 3 min in 100% xylene, 2 × 3 min in 100% ethanol, 1 × 3 min in 95% ethanol, 1 × 3 min in 70% ethanol, 1 × 3 min in 50% ethanol, and 1x in water. For heat-induced epitope retrieval, slides were cooked in 10 mM sodium citrate (pH 6.0) containing 0.1% Tween in a microwavable vessel for 20 min at 900 W, and subsequently cooled gently and rinsed in water and PBS containing 0.1% Tween. Tissue sections on slides were demarcated with an ImmEdge pen (Vector Laboratories, Peterborough, UK). For proteinase-induced epitope retrieval (for lysozyme staining), slides bearing tissue sections were demarcated with ImmEdge pen. Each section was incubated in a drop of 50 mM Tris pH 7.5 containing 1% v/v proteinase K (Qiagen) for 5 min at room temperature (RT) in a humid chamber. Slides were then washed twice in PBS containing 0.1% Tween.

For antibody staining, slides were treated with peroxidase-blocking reagent (Dako, Carpinteria CA, USA) for 10 min and washed 3x in PBS containing 0.1% Tween for 5 min. Slides were then blocked in PBS containing 10% BSA for 30 min at RT in a humid chamber, incubated with primary antibody in PBS containing 10% BSA for 90 min at RT (or overnight at 4 °C), and subsequently washed 3x for 5 min with PBS containing 0.1% Tween. For detection, the DAB detection kit (Dako, α-lysozyme, α-phospho-histone H3, pH3) or Polymer Refine Detection System (Leica, α-BrdU) was used; BrdU detection was carried out at the Histopathology and in situ Hybridization Facility (CRUK Cambridge Institute). Hematoxylin (25% solution in water, Dako) was used for counterstaining (for 5 min), followed by two wash steps in water. For TUNEL staining, the DeadEnd Colorimetric Apoptosis Detection System (Promega, Madison WI, USA) was used. For PAS-D staining, slides were incubated as follows: 2% amylase (Sigma Aldrich) solution in ultra-pure water for 15 min at 37 °C; tap water for 30 s; ultra-pure water for 30 s; 1% Alcian blue 8GX (TCS Biosciences, Buckingham, UK) in 3% acetic acid for 10 min; ultra-pure water for 30 s; 0.5% periodic acid (VWR, Radnor PA, USA) in ultra-pure water for 5 min; ultra-pure water for 1 min; Schiff’s reagent (TCS Biosciences) for 20 min; ultra-pure water for 1 min; tap water for 15 min; Harris Haematoxilin (CellPath, Newtown, UK) for 15 s; tap water for 1 min; 0.3% hydrochloric acid in 70% ethanol for 5 s; tap water for 1 min; 0.3% sodium borate for 15 s; tap water for 1 min; 3x ethanol for 30 s; 3x xylene for 30 s. Staining was done with Gemini AS (ThermoFisher Scientific) at the Human Research Tissue Bank, supported by the NIHR Cambridge Biomedical Research Centre. For detection of γ-H2AX, a commercial antibody (Cell Signalling Technology, Danvers MA, USA) was applied on an automated immunostainer (Bond-III, Leica Biosystems), using a working dilution of 1:50. Antigen retrieval was performed using the Bond Epitope Retrieval 2 Solution (Leica Biosystems) for 20 min. The Bond Polymer Refine Detection kit (Leica Biosystems) was used for visualizing antigens. All IHC slides were dehydrated by the following incubations: 1 × 3 min in 50% ethanol; 1 × 3 min at 70% ethanol; 1 × 3 min at 95% ethanol; 2 × 3 min at 100% ethanol; 2 × 3 min at 100% xylene. They were subsequently air-dried and mounted in Glycergel Mounting Medium (Dako). Imaging was done with a Zeiss microscope and Zeiss AxioCam MRc5 camera.

For IF, incubation with primary antibodies and washes were done as described above for IHC. Slides were subsequently incubated with fluorescently-labeled secondary antibodies for 90 min at RT in a dark humid chamber, washed 3x for 5 min with PBS containing 0.1% Tween and mounted in Vectashield Antifade Mounting Medium with DAPI (Vector Laboratories, no. H-1200). Images were acquired at identical settings with a Zeiss Confocal Microscope.

Cells positive for TUNEL, γ-H2AX and pH3 were counted manually with a cell counter. Cells positive for lysozyme or PAS-D, and total number of adenoma cells, were counted using CellProfiler software (cellprofiler.org). The adenoma surface areas were determined using FIJI software (fiji.sc).

Wild type and CRISPR clones of SW480 cells were imaged with a Zeiss Confocal Microscope in 8 well glass bottom µ-slides (Ibidi, Martinsried, Germany) after being stained with α-BCL9-2 (R & D Systems) and α-β-catenin (BD) primary antibodies, donkey α-sheep and goat α-mouse secondary antibodies (Life Technologies), and Hoechst stain.

### CRISPR/Cas9 genome editing

Genome editing in SW480 cells was essentially performed as previously described^69^, using single guide RNA-encoding plasmid derivatives of pSpCas9(BB)−2A-GFP (PX458). Cells were selected for high expression of GFP by fluorescence-activated cell sorting (FACS) 48 h post-transfection, and sorted at a density of 1 cell well^−1^ in a 96-well plate, to isolate single clones for subsequent screening by western blot analysis and DNA sequencing. For the proliferation assay (Supplementary Figure 4), GFP-positive cells were sorted in bulk at a density of 10^3^ cells well^−1^ in a 96-well plate, and the cell density at subsequent days was measured using an EC800 flow cytometer (Sony Biotechnology, Weybridge, UK).

### RNA extraction

Frozen adenomas and crypt samples were thawed on ice in RLT buffer (Qiagen, Hilden, Germany) containing 1% β-mercaptoethanol, and homogenized with a Qiagen TissueRuptor followed by QIAshredder columns according to the manufacturer’s instructions. RNA was extracted from tissue lysates with the RNeasy Kit, and treated with RNase-free DNase I (Qiagen). Typically, 2–3 RNA extractions from one mouse was pooled for a single sample. RNA concentration and quality were determined with a NanoDrop 2000 (Thermo Scientific, Waltham MA, USA) and a 2100-Bioanalyzer (Agilent Technologies, Santa Clara CA, USA).

### Gene expression analysis

Two batches of quality-controlled RNA samples were generated (Supplementary Table 1), for microarray analysis by Cambridge Genomic Services (University of Cambridge). RNA was linearly amplified with the Illumina Totalprep RNA Amplification Kit, and the resulting cRNA was hybridized to the MouseWG-6 v2.0 Expression BeadChip (Illumina, San Diego CA, USA) overnight; subsequently, the BeadChip was washed, stained, and scanned with the Illumina BeadArray Reader. Sample and control probe profiles were exported from *GenomeStudio software v2011.1* (Illumina) without background correction or normalisation, and subsequently loaded into the R environment. Relevant subsets of the data were imported separately for each comparison into a LumiBatch object, and filtered by keeping probes that were detected in at least one sample (using a detection *p*-value < 0.01). The data were then quality-controlled, transformed (Variance Stabilising Transformation, VST) and quantile-normalised with the *lumi v2.30.0* package^70^, and non-biological experimental variation between the two microarray sample batches was eliminated with the ComBat function from the *SVA v3.26.0* package. Differentially expressed genes were identified with the *limma v3.34.9* package^71^, and *p*-values were corrected for using the Benjamini & Hochberg False Discovery Rate (FDR). Throughout our study, an FDR-adjusted *p-*value of *p* < 0.01 was used as the threshold for statistical significance unless otherwise stated.

For RNA-seq analysis, quality-controlled RNA from each sample was used to generate RNA-seq libraries with the NEBNext Ultra II Directional RNA Library Prep Kit (New England Biolabs, Ipswich MA, USA) according to the manufacturer’s instructions. Briefly, mRNA was isolated from 1 μg of total RNA from each sample. RNA was fragmented enzymatically for 15 min at 94 °C and used to generate cDNA libraries with an average size of 361 bp ± 12 bp (SD) barcoded with NEBNext Multiplex Oligos for Illumina Set 1 (New England Biolabs). Sequencing was subsequently performed on one lane of an Illumina HiSeq 4000 at the CRUK Cambridge Institute Genomics Core to generate ~30 × 10^6^ single-end stranded 50 base pair reads per sample. Reads for each sample were demultiplexed according to the barcodes, and transcript-level abundances were quantified by quasi-mapping the reads against an index build from the GRCm38 transcriptome using *Salmon v0.9.1*^72^. These quantifications were subsequently imported into the R environment using the *tximport v1.6.0* package^73^, and differentially expressed genes were identified using the *DEseq2 v1.18.1* package^74^. An FDR-adjusted *p* < 0.01 was used as a threshold for statistical significance during subsequent analysis. Quantitative set analysis for gene expression (QuSAGE)^75^ was performed on rlog transformed read counts using previously published Wnt and stem cell marker gene sets (see Supplementary Figure 9, for references).

### Reagents and resources

The reagents and resources used in this study are listed in Supplementary Table 2.

## Supplementary information

Supplementary Information

## Data Availability

The microarray and RNA sequencing data that support the findings of this study are available in NCBI’s Gene Expression Omnibus and are accessible through GEO Series reference GSE121145.
