# Supplementary material for: Bcl9 and Pygo synergise downstream of Apc to effect intestinal neoplasia in FAP mouse models
Source: Nat Commun. 2019 Feb 13;10:724. doi: 10.1038/s41467-018-08164-z (PMC6374407; doi:10.1038/s41467-018-08164-z)
Supplement: Supplementary file 1 — Supplementary Information [file 41467_2018_8164_MOESM1_ESM.pdf]

SUPPLEMENTARY INFORMATION

Bcl9 and Pygo synergise downstream of *Apc* to effect intestinal neoplasia in FAP mouse models

Mieszczanek et al.

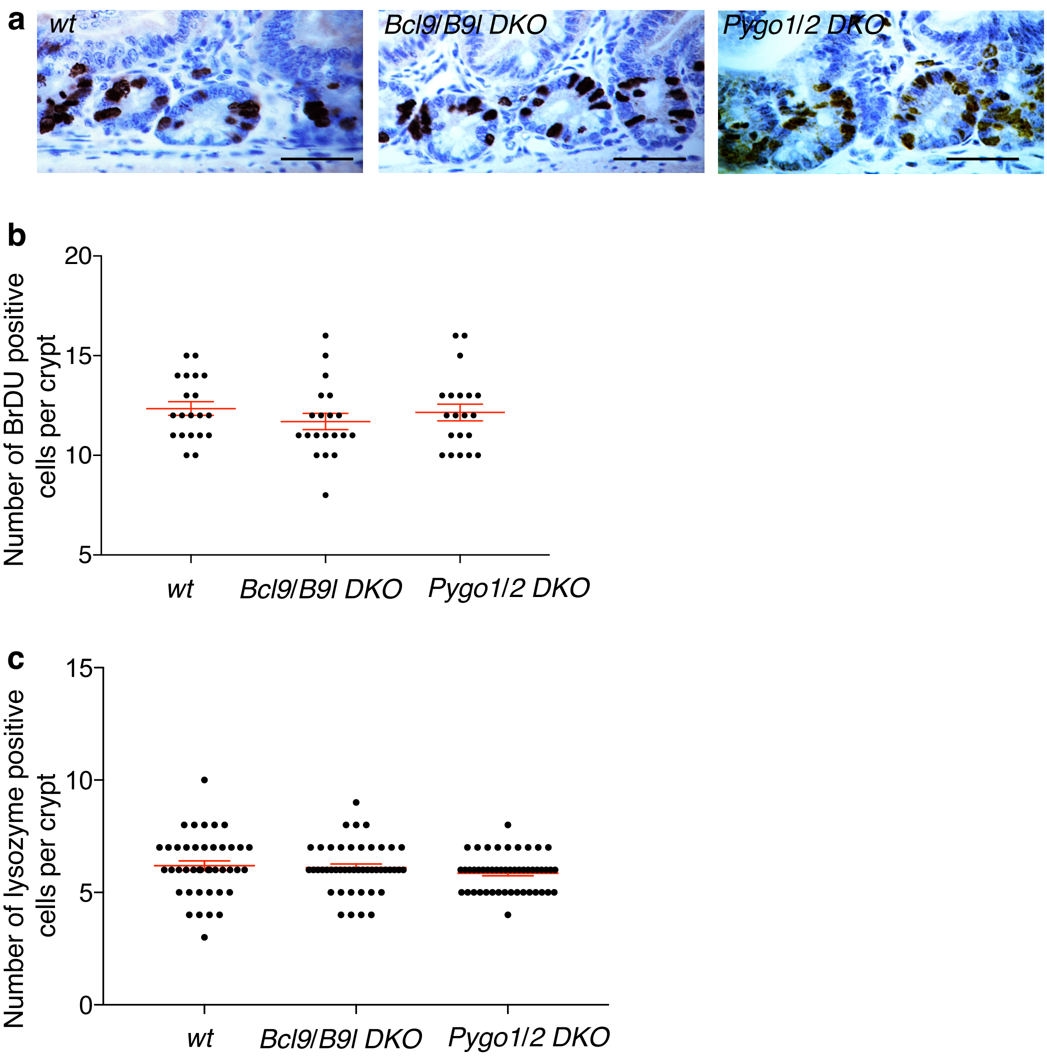

Supplementary Figure 1. The numbers of proliferative crypt cells are not affected by loss of Bcl9 or Pygo. (**a**) Representative cross-sections through crypts from small intestines (genotypes indicated in panels), after fixation and staining with a-BrdU antibody (counterstained with hematoxylin); scale bar, 100 µm. (**b, c**) Numbers of (**b**) BrdU-positive and (**c**) lysozyme-positive cells (marking dividing and Paneth cells, respectively) in cross-sections of normal (*wt*) and *DKO* crypts (selected on diameter ~ 100 mm, and near-circular orientation in sectional plane), as indicated underneath graphs; n = 20 (2 x 10 crypts, from 2 mice) per cohort; mean, and standard error of the mean (SEM) are indicated by horizontal lines; no statistically significant differences were found.

0	1.7	0	2	0.7	0.1	*Galt*
0	1.3	0	1.7	0.2	0.2	*Ccnyl1*
0.2	1.3	0.3	0	0.1	0.6	*4930463P07Rik*
0	1.3	1.3	0.1	0.3	0.4	*Prdm16*
0	1.7	0.1	0.6	0	0.3	*Papss2*
0	1.5	4	0.8	1.6	1.2	*Mdm2*
0.8	1.5	1.8	0.7	0.9	0.3	*Oas1g*
0.1	1.7	2.3	0	0.2	0	*Rpl23*
0.1	1.7	2.2	0	0.7	0.5	*Nt5e*
0	1.3	2.3	1.3	0	0.1	*Srr*
0.1	1.7	0.1	0.4	0.4	0.4	*6330442E10Rik*
0.8	1.5	0.7	0.9	0.6	1	*Oasl1*
0.1	1.4	1.2	0.5	1.6	1	*2010107G12Rik*
0.1	1.5	0.1	0.5	2	0.8	*Gpd2*
0	1.5	1.7	1.8	2.4	0.6	*Creb3l2*
0.3	1.4	0.7	1.9	1.9	0	*2010109I03Rik*
0.2	1.3	4.9	0.7	1.1	0.1	*Tuft1*
0	1.3	5.4	0.4	0.1	0.8	*Rfx2*
0	1.3	2.8	1.1	0.1	0.2	*Hist1h1c*
0.3	1.7	4.4	0.6	0.3	0.1	*Abhd2*
0	1.3	4.5	0	0.5	0.9	*Klf4*
0	1.5	5.2	2.5	0.5	0.1	*Cdr2*
0	1.4	3.2	1.4	0.7	0.3	*Mme*
0.2	1.5	2.8	1.3	1.1	0.5	*Cidec*
0	1.7	0.1	1.4	1.3	0	*Ncoa4*
0	1.5	1.5	3	1.3	0.4	*Idh1*
0.7	1.5	3.8	4.4	3.5	0.1	*Chgb*
0	1.3	4.7	2.7	0	0.2	*Cyp3a13*
0	1.4	4.2	2.1	1.6	0.8	*Defcr-rs2*
0	1.7	0	0	0	0.7	*AY761185*
0	1.6	0	1.1	0	1.7	*Defcr-rs10*
0	2.6	0	1.9	1.2	0.6	*LOC100044289*
0	1.7	0	1.8	0	0.7	*Defcr-rs7*
0	2.2	0	2.6	1.9	8.5	*Defcr4*
0	1.7	4	3.1	3.5	6.7	*Defa1*
0	1.8	0.5	3.2	3.4	0	*Clca4*
0	1.3	0.8	2	2.8	1.2	*Ccl9*
0	1.4	4.1	1.6	1.8	1.6	*Defcr26*
0	1.4	4.9	0.3	0.9	0.8	*Defcr21*
0	1.3	4.8	0.1	0.7	0.1	*Cdca8*
0	1.3	5.7	0.1	1.4	0.3	*Prpf19*
0	1.3	4.8	0.7	1	0.1	*LOC381330*
0	1.4	5.4	0.7	0.7	0.1	*Nup210*
0	1.4	4.9	0.5	0.5	0.5	*EG666609*
0	1.4	4.3	0.4	0.2	0.3	*Hist1h2ag*
0	1.3	4.2	0.3	1	0.7	*Imp3*
0	1.4	4.3	0.2	1	0.1	*Rpl31*
0	1.3	6.5	0.3	0.3	0.1	*LOC665237*
0	1.4	3.9	0.4	0.7	1.2	*Lrig1*
0	1.3	4	0.3	0.3	0.6	*Pbk*
0	1.6	3.5	0.2	0.9	1.1	*LOC100042405*
0	1.5	3.1	0.2	0.6	0.8	*Hist1h2ad*
0	1.3	5.1	0.1	0.2	0.4	*Hist1h2af*
0	1.4	4.2	0	0.1	0	*Hist1h2an*
0	1.3	5.9	0.1	0.6	0.1	*Hist2h2ac*
0	1.4	6	0.3	0.2	0.3	*EG243642*
0	1.5	5.3	0.1	0.2	0.1	*Rps26*
0	1.4	5.9	1.8	1.4	0.4	*LOC100046853*
0	1.7	6.5	1.8	0.9	0	*LOC380692*
0	1.3	5	1.6	0.8	0.3	*Nol5*
0	1.5	4	1.8	0	0.2	*Hmga1*
0	1.4	5.8	1.2	1.1	0.5	*Impdh2*
0.1	1.4	4.4	0.1	1	0.2	*Tgm2*
0	1.3	7.1	0.1	2.2	0.5	*Fads1*
0	1.6	7.1	1.2	0	0.7	*Tubb5*
0	1.4	6	4.2	1.2	0.3	*Paox*
0.2	1.5	6.9	3.8	0.4	0.2	*Phlda1*
0	1.3	5.9	2.4	0.6	1.1	*Habp2*
0	1.4	3.6	2.5	0.1	0.3	*Prelp*
0	1.3	8.2	1.3	3	0	*Ifitm2*
0	1.3	4.4	2.9	1.8	0.4	*Rcl1*
0	1.3	4.2	3	1.6	0.5	*Eef1b2*
0	1.3	2.4	0.8	0.6	0.2	*LOC382061*
0	1.3	4.2	2.1	0.9	0.2	*Rpl36a*
0	1.7	1	0.6	0.4	0.1	*Nusap1*
0	1.3	1.8	1.1	1.8	0.5	*5730427N09Rik*
0	1.3	0.1	2.2	1.2	0.5	*2310039H08Rik*
0	1.5	1.6	0.1	0.4	1	*LOC100047998*
0	1.3	1	0	0.3	0	*Sgol1*
0	1.3	2.9	0.3	0.2	0.1	*Rps12*
0	1.4	4.6	0.4	0.1	0.4	*Krtcap2*
0	1.6	3	0.4	2.3	1.9	*Slc12a2*
0	1.4	6.5	0.2	0.3	0	*Cfl2*
0	1.7	1.9	1.9	0.1	0.3	*Bmp3*
0	1.4	2.2	3	0.4	0.7	*LOC100046056*
0	1.6	0.7	3.4	3	2.4	*Lars2*
0	1.7	2	1.3	0	3.8	*Pygo2*
0	1.5	2.8	1.9	3.4	8.7	*Dapk2*
0	1.7	2.7	2.5	0.8	4.2	*2810417H13Rik*
0	1.3	1.3	4.8	0.2	2	*Kif23*
0.1	1.4	0.6	0	2.5	0	*LOC226685*
2.4	0.1	2.1	3	3.9	0.2	*Amica1*
2.4	0.1	1.4	2.8	4.6	0.6	*LOC270152*
1	1.5	6.3	0.4	2.4	2.7	*2010204N08Rik*
2.7	0.7	6.8	4.9	4.1	0.2	*Tmem25*
0.1	1.5	5.2	1.7	1	0.1	*Gcg*

Bcl9/B9l DKO vs *wt* crypts (*villin.Cre/+*)

Pygo1/2 DKO vs *wt* crypts (*villin.Cre/+*)

Apc^{Min} adenomas vs *wt* crypts

Apc^{Min} *QKO* adenomas vs *Apc*^{Min} adenomas

Apc^{Min} *Bcl9/B9l DKO* adenomas vs *Apc*^{Min} adenomas

Apc^{Min} *Pygo1/2 DKO* adenomas vs *Apc*^{Min} adenomas

-4 -2 0 2 4

Log2 fold change

Supplementary Figure 2. Changes of gene expression profiles in *DKO vs* control crypt samples. List of all significant gene expression changes in *DKO* vs wt crypt preparations (*left two columns*); for comparison, 4 other cohorts are shown (color-coded as in main **Figure 2b**); for genotypes, see key on the right; red and blue shades represent log2 fold changes (color-coded as in key on the right); numbers in squares signify -log10 FDR-adjusted *p*-values. Histone H2A (*pink underlay*) and ribosomal protein genes (*green underlay*) show opposite regulatory trends from Defensin genes (*yellow underlay*) in *DKO* vs wt crypts, and trend reversals in Apc^{Min} adenomas vs wt crypts*.* Note also that Defensin gene probes have a tendency to cross-hybridize (e.g. *Defa1* and *Defa5*).

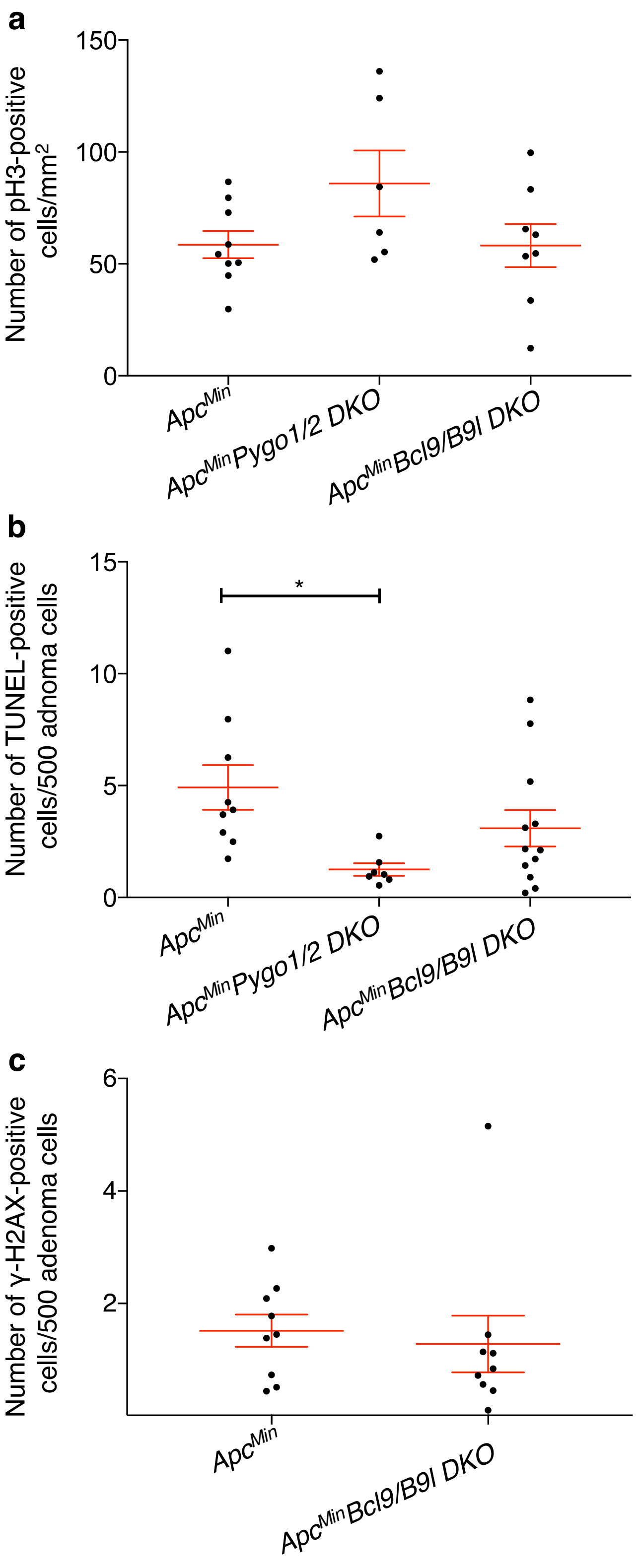

Supplementary Figure 3. Mitotic, apoptotic and g-H2AX-positive cells in *DKO* and control *Apc*^{Min} adenomas. Quantitations of cells positive for (**a**) pH3, (**b**) TUNEL or (**c**) g-H2AX, as indicated on the left, in *Apc*^{Min} *DKO* and control adenomas, as indicated underneath graphs. Statistical significance, *, $p < 0.02$ (Tukey's multiple comparisons test).

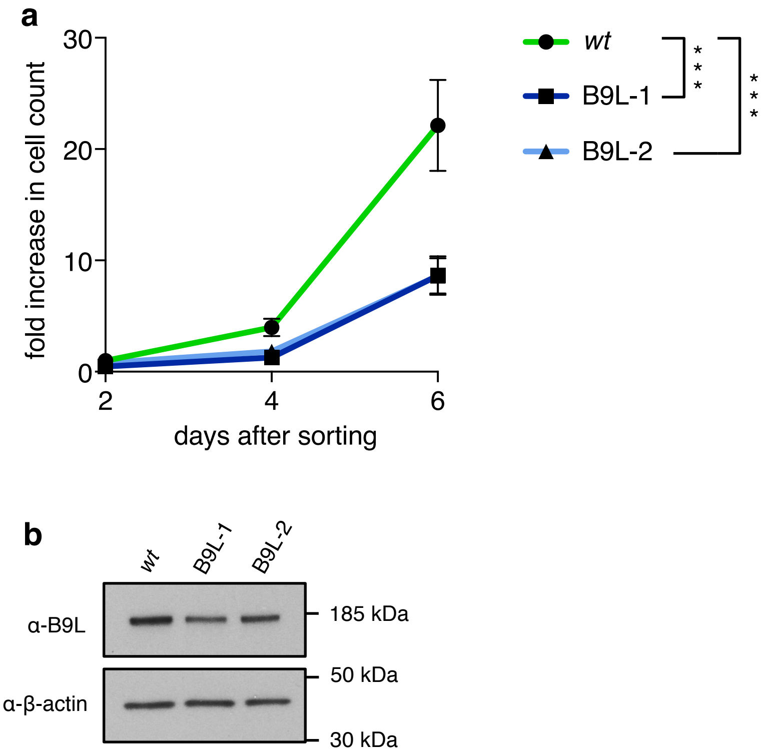

c

sgRNA	Sequence (5′-3′)
B9L-1	(G)TTGAGTTGCTAGGCGAGATC
B9L-2	GTGCTCAGGGGTAGCGGTTC

Supplementary Figure 4. B9L depletion of SW480 colorectal cancer cells by CRISPR-engineering. **(a)** Proliferation assay of B9L-depleted and matched parental SW480 cells over 2-6 days (the period of exponential growth), following sorting of individual cells (which resulted in >95% of cell singlets across each experimental group); n=3 independent experiments, $p < 0.001$ (two-way ANOVA, with Dunnett's multiple comparisons test); the fold increase in cell numbers is relative to the cell numbers of matched parental SW480 cells at day 2. Note that we also attempted complete deletion of B9L from SW480 colorectal cancer cells by CRISPR engineering, but this process consistently yielded multi-nucleate cells that flattened out, and it proved impossible to recover any B9L-deleted growing colonies. **(b)** Western blot of B9L-depleted and matched parental SW480 cells; to obtain sufficient material, cells were harvested 10 days after sorting, and so the observed B9L reductions are likely to be underestimates. **(c)** Sequences of single guide RNAs used for CRIPSR engineering.

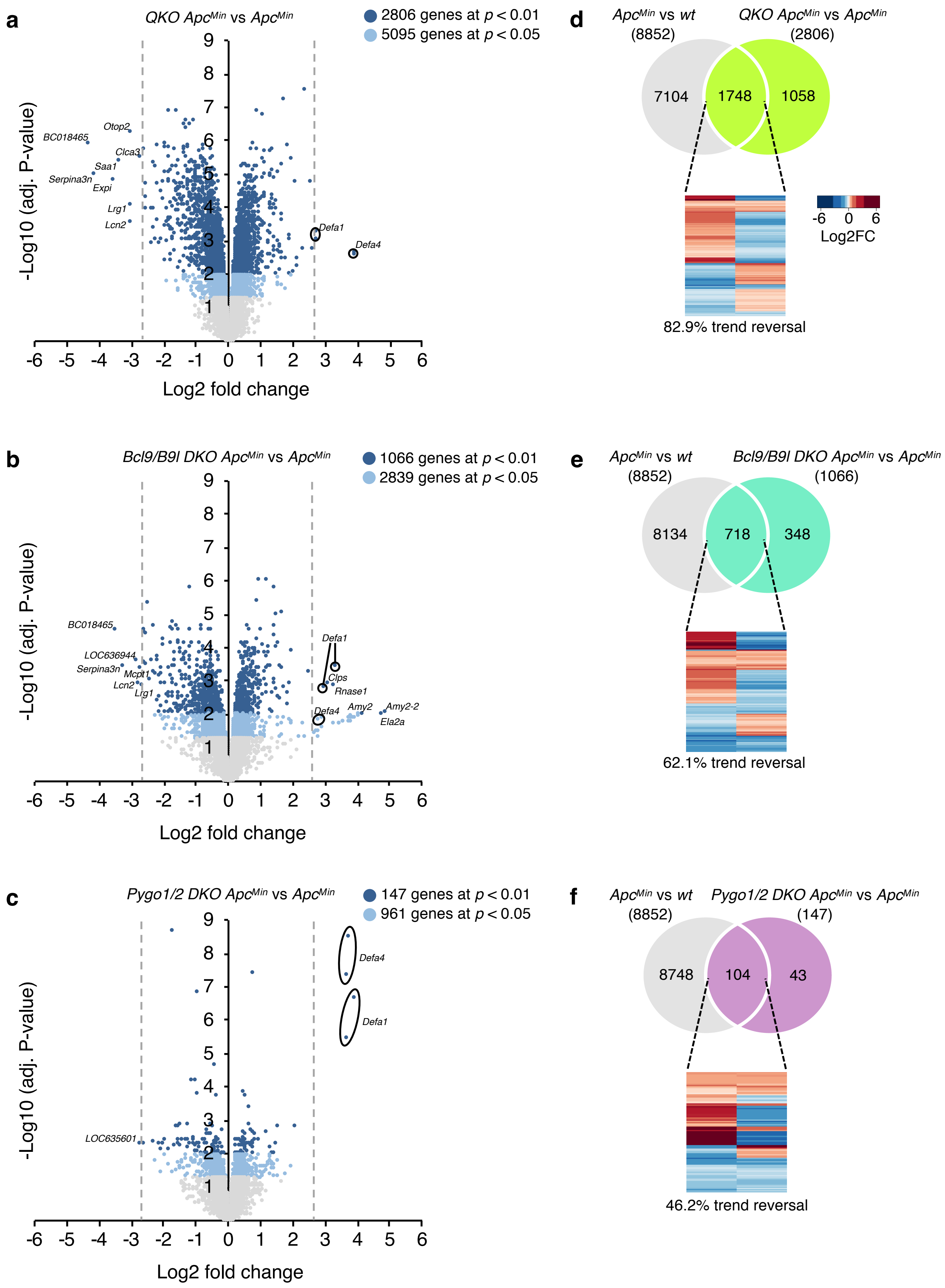

a
QKO ApcMin vs ApcMin
2806 genes at p < 0.01
5095 genes at p < 0.05
-Log10 (adj. P-value)
Log2 fold change
BC018465
Otop2
Clca3
Saa1
Serpina3n
Expi
Lrg1
Lcn2
Defa1
Defa4
b
Bcl9/B9l DKO ApcMin vs ApcMin
1066 genes at p < 0.01
2839 genes at p < 0.05
BC018465
LOC636944
Serpina3n
Mcpt1
Lcn2
Lrg1
Defa1
Clps
Rnase1
Defa4
Amy2
Amy2-2
Ela2a
c
Pygo1/2 DKO ApcMin vs ApcMin
147 genes at p < 0.01
961 genes at p < 0.05
LOC635601
Defa4
Defa1
d
ApcMin vs wt
(8852)
QKO ApcMin vs ApcMin
(2806)
7104
1748
1058
Log2FC
82.9% trend reversal
e
ApcMin vs wt
(8852)
Bcl9/B9l DKO ApcMin vs ApcMin
(1066)
8134
718
348
62.1% trend reversal
f
ApcMin vs wt
(8852)
Pygo1/2 DKO ApcMin vs ApcMin
(147)
8748
104
43
46.2% trend reversal

Supplementary Figure 5. Changes of gene expression profiles in *DKO* vs control *Apc*^{Min} adenomas. (**a-c**) Volcano plots showing all significant gene probe changes in *DKO* or *QKO* adenomas vs *Apc*^{Min} control adenomas (see also main **Figure 2**), with the most up- or down-regulated genes labeled (including the Defensin genes *Defa1* and *Defa4*); *dark blue*, $p < 0.01$; *light blue*, $p < 0.05$. (**d-f**) Venn diagrams showing number of gene probe changes in cohorts as indicated above; *underneath*, heat-maps of shared changes between two cohorts (*red*, upregulated; *blue*, downregulated); *right*, key for log2 fold change; *underneath*, percentages of trend reversals in these gene probe sets shared between the two cohorts.

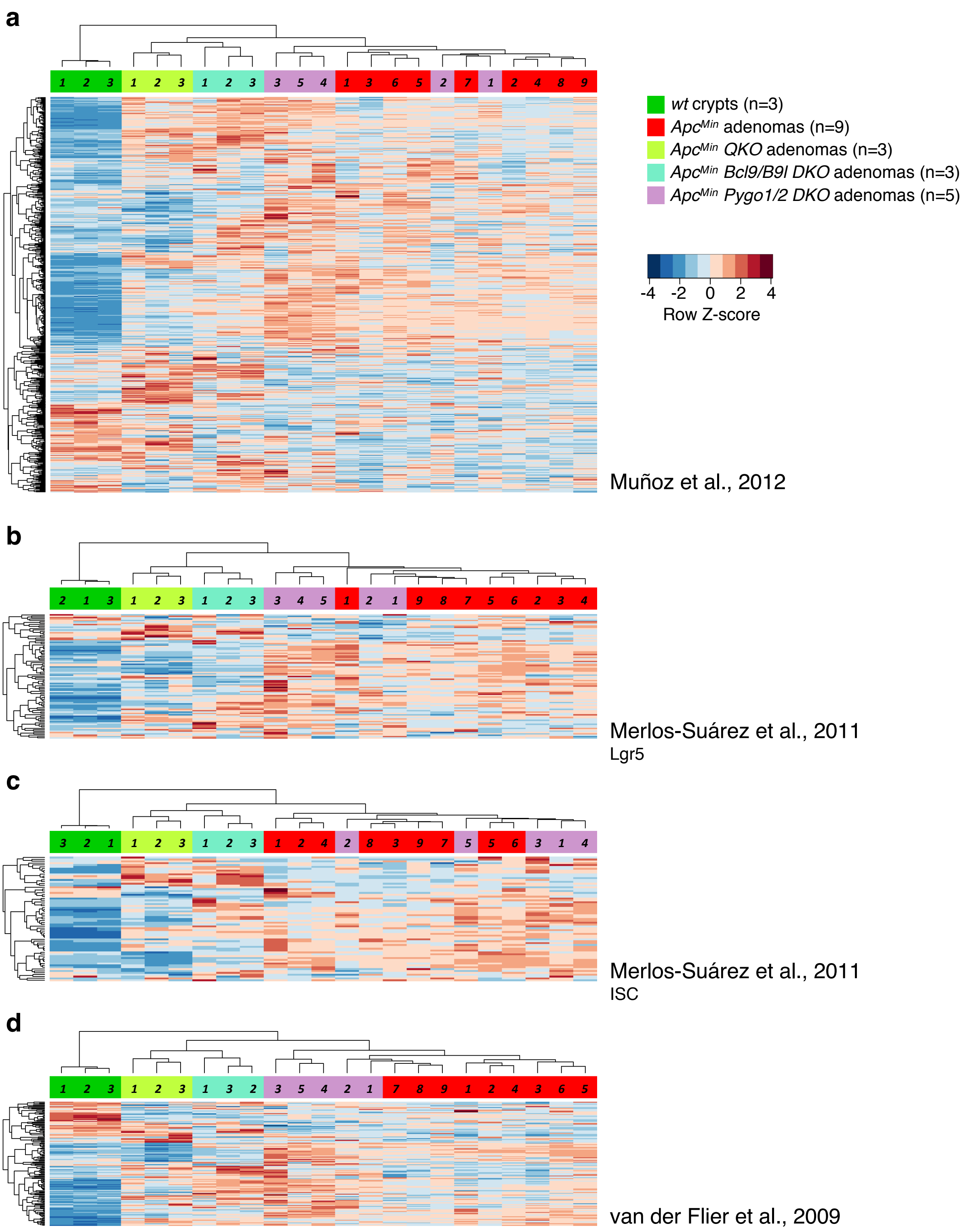

Supplementary Figure 6. Changes of gene expression profiles for intestinal stem cell signatures across cohorts. Heat-map showing hierarchical clustering of gene changes (p < 0.01) representing previously determined stem cell signatures (*right*; see also main text and References) across 5 different cohorts, as indicated in bars (color-coding and designations of individual samples as in main **Figure 2**); color indicate Z-scores of normalised, VST- and batch-adjusted expression values for each individual gene probe (*red*, high expression; *blue*, low expression).

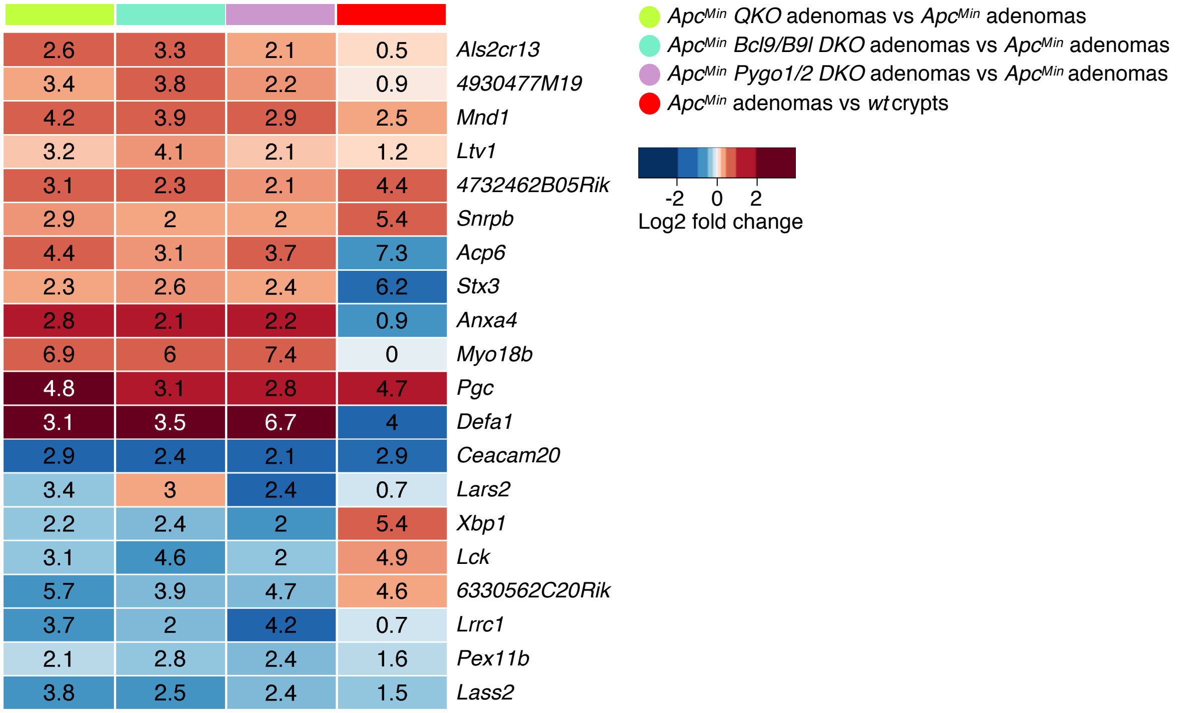

Supplementary Figure 7. Gene expression changes in *Apc^{Min}* adenomas shared between all four cohorts. List of gene expression changes ($p < 0.01$) shared between 3 cohorts, as in main **Figure 3b**, with genotypes shown in key on the right (color-coding as in main **Figure 3**); red and blue shades represent log2 fold changes (color-coded as in key, *right*); numbers in squares signify -log10 FDR-adjusted *p*-values; *right*, gene names (for *Defa1* and *Lass2*, referring to the probe showing highest significance).

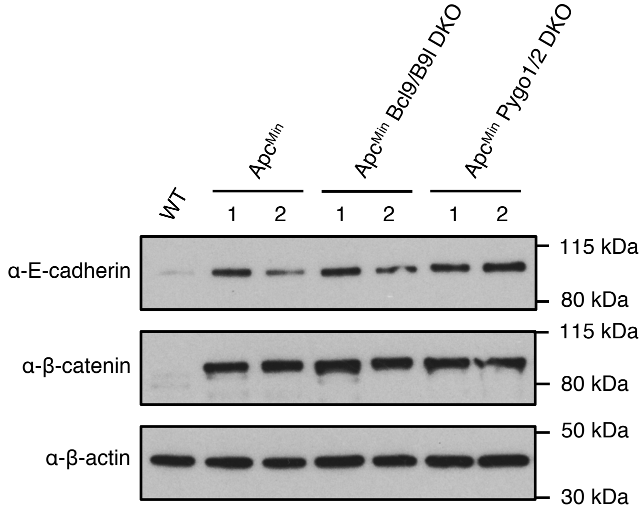

Supplementary Figure 8. Levels of b-catenin and E-cadherin in *DKO* and *Apc^Min^* control adenomas. Western blots of total cell lysates from normal crypt samples, and from *DKO* and *Apc^Min^* adenoma samples (from two different mice each, as indicated by numbers above lanes), probed with antibodies as shown on the left. We noted some variability between individual samples, owing to their preparation (based on mechanical scraping, rather than chelation of Mg^{++}, to preserve E-cadherin-based adhesion complexes; see Methods), however, b-catenin and E-cadherin were consistently upregulated in adenomas compared to normal crypts across several matched sample pairs (n = 9), likely owing to post-transcriptional co-stabilisation (as their RNA levels were not induced in the adenomas; **Figure 2a**): b-catenin is stabilised owing to *Apc* loss, whereas E-cadherin may be co-stabilised by the high levels of cytoplasmic b-catenin in these cells, owing to the mutual association of these proteins upon translation[1]. However, we did not detect any consistent changes in the levels of b-catenin and E-cadherin between *DKO* and control adenomas.

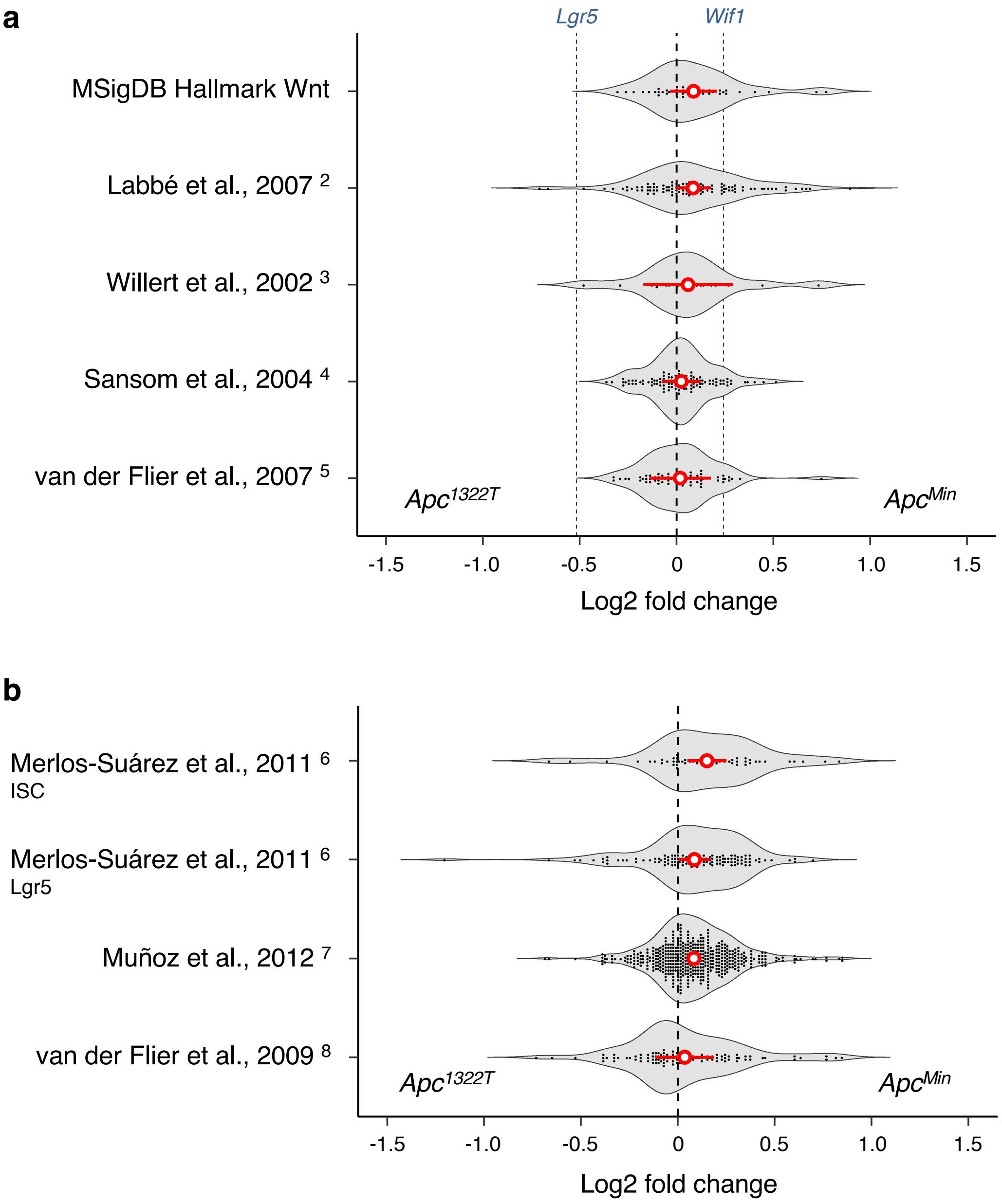

Supplementary Figure 9. Comparisons of RNA expression profiles in Apc^{Min} and Apc^{1332T} adenomas. Violin plots based on QuSAGE analysis of RNA expression changes in Apc^{Min} vs Apc^{1332T} adenomas regarding previously determined sets of (**a**) Wnt and *Apc* target genes or (**b**) intestinal stem cell signature genes, as indicated by the references (*left*); MSigDB Hallmark Wnt, broadly validated collection of Wnt target genes (GSEA, Broad Institute). Red circles and bars represent mean values of log2 fold changes and SEM, respectively; blue dotted lines indicate log2 fold changes determined for downregulation of *Lgr5* and upregulation of *Wif1* in our data set, previously identified as the two Wnt target genes showing the most significant expression changes (based on RT-qPCR) between the two types of adenomas[9]. Note the overall trends of Wnt target and stem cell gene sets to be more upregulated in Apc^{Min} than Apc^{1332T} adenomas.

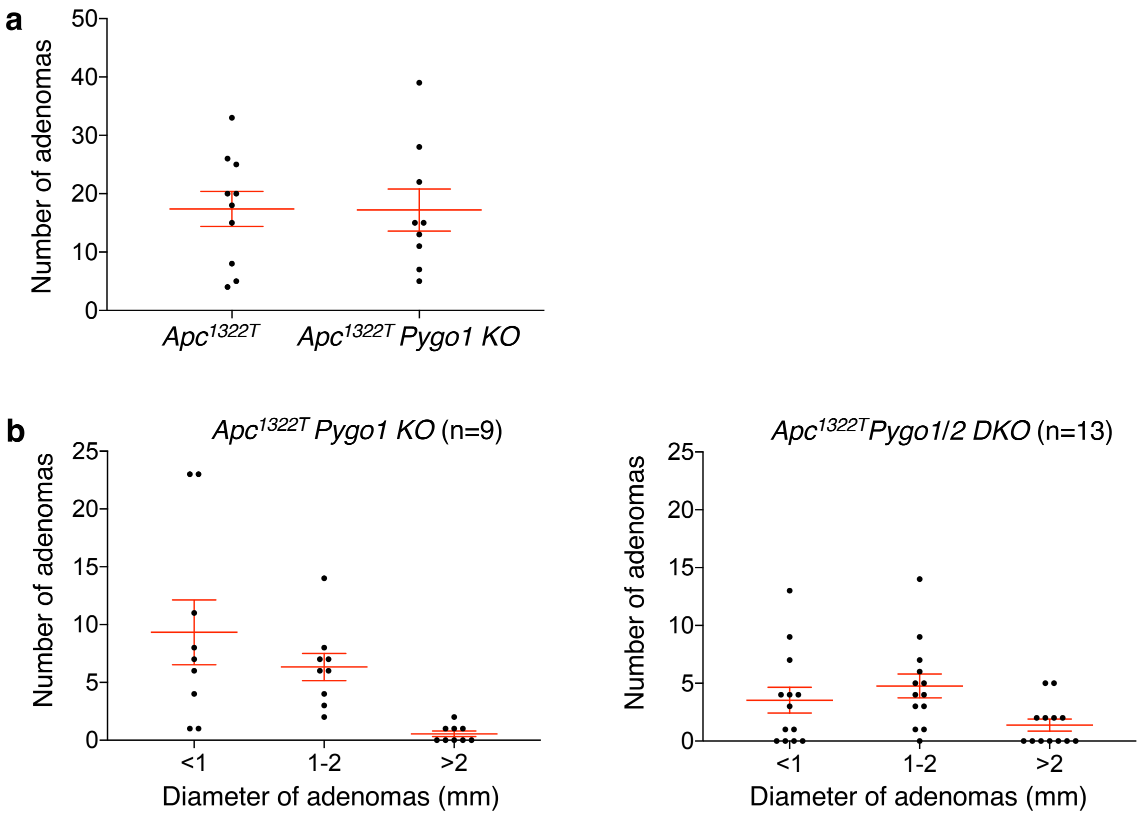

Supplementary Figure 10. Tumor numbers and size distributions in *Apc*^{1332T} *Pygo1/2 DKO* and littermate controls. (**a**) Adenoma counts in small intestines from 77 day-old *Apc*^{1332T} *Pygo1/2 DKO* mice and their *Pygo1 KO* littermates, as indicated underneath graph; each dot represents one mouse; mean and SEM indicated by horizontal lines; no significant differences between the two cohorts were observed (Tukey's multiple comparisons test). (**b**) Tumors shown in (**a**) were grouped into three size classes, as indicated underneath graphs (see also main text), and counted; horizontal lines as in (**a**).

Supplementary Table 1. RNA profiling samples.

Sample batch	Mouse genotype	Sample numbers	Total number of samples	Platform
Microarray batch 1	wt (C57BL/6J)	1, 2, 3	3	MouseWG-6 v2.0
Microarray batch 1	wt (C57BL/6J, villin.Cre/+)	1, 2, 3	3	MouseWG-6 v2.0
Microarray batch 1	Bcl9/B9l DKO ($Bcl9^{LoxP/LoxP}$, $B9l^{LoxP/LoxP}$, villin.Cre/+)	1, 2, 3	3	MouseWG-6 v2.0
Microarray batch 1	Pygo1/2 DKO ($Pygo1^{-/-}$, $Pygo2^{LoxP/LoxP}$, villin.Cre/+)	1, 2, 3	3	MouseWG-6 v2.0
Microarray batch 1	Apc^{Min}/+	1, 2, 3, 5, 7, 9	6	MouseWG-6 v2.0
Microarray batch 1	Apc^{Min}/+ Bcl9/B9l DKO ($Bcl9^{LoxP/LoxP}$, $B9l^{LoxP/LoxP}$, villin.Cre/+)	1, 2, 3	3	MouseWG-6 v2.0
Microarray batch 1	Apc^{Min}/+ Pygo1/2 DKO ($Pygo1^{-/-}$, $Pygo2^{LoxP/LoxP}$, villin.Cre/+)	1, 2, 3, 4, 5	5	MouseWG-6 v2.0
Microarray batch 2	Apc^{Min}/+ QKO ($Bcl9^{LoxP/LoxP}$, $B9l^{LoxP/LoxP}$, $Pygo1^{-/-}$, $Pygo2^{LoxP/LoxP}$, villin.Cre/+)	1, 2, 3	3	MouseWG-6 v2.0
Microarray batch 2	Apc^{Min}/+	4, 6, 8	3	MouseWG-6 v2.0
RNA sequencing	Apc^{Min}/+	10, 11, 12	3	SE50bp, Illumina HiSeq 4000
RNA sequencing	Apc^{1322T}/+	1, 2, 3	3	SE50bp, Illumina HiSeq 4000

Supplementary Table 2. Reagents and resources.

Reagent or resource	Source	Identifier
α-β-catenin	BD Transduction Laboratories	Cat#610153
α-BCL9-2	R & D Systems	Cat#AF4967
α-E-Cadherin (24E10)	Cell Signalling Technology	Cat#3195
α-β-actin	Sigma Aldrich	Cat#A5441
α-BrdU	Abcam	Cat#ab1893
α-lysozyme	Dako	Cat#A0099
α-Phospho-Histone H3 (Ser28)	Cell Signalling Technology	Cat#9713
α-Phospho-Histone H2A.X (Ser139)	Cell Signalling Technology	Cat#2577
Rabbit α-Sheep	Jackson ImmunoResearch	Cat#313-005-003
Alexa Fluor 488 conjugated Goat α-Rabbit	Life Technologies	Cat#A11008
Alexa Fluor 488 conjugated Donkey α-Sheep	Life Technologies	Cat#A11015
Alexa Fluor 546 conjugated Goat a-Mouse	Life Technologies	Cat#A11003
HRP conjugated Goat α-Mouse	Santa Cruz Biotechnology	Cat#sc-2005
HRP conjugated Goat α-Rabbit	Santa Cruz Biotechnology	Cat#sc-2301
8 well glass bottom μ-slides	Ibidi	Cat#80827
DeadEnd Colorimetric Apoptosis Detection System (TUNEL)	Promega	Cat# G7360
Proteinase K	Qiagen	Cat#19133
Amylase	Sigma Aldrich	Cat#A3176-1MU
Alcian blue 8GX	TCS Biosciences	Cat#HS116-500
ImmEdge Hydrophobic Barrier Pen	Vector Laboratories	Cat#H-4001
VECTASHIELD Mounting Medium with DAPI	Vector Laboratories	Cat#H-1200
Bond Epitope Retrieval 2 Solution	Leica	Cat#AR9640
Bond Polymer Refine Detection kit	Leica, Biosystems	Cat#DS9800
DAB detection kit	Dako	Cat#K4010
Polymer Refine Detection System	Leica	Cat#DS9800
Harris Haematoxilin	Cellpath	Cat#RBA-4205-00A
Hematoxylin	Dako	Cat#S3309
Glycergel Mounting Medium	Dako	C056330-2
TaqMan Sample-to-SNP Kit	ThermoFisher Scientific	Cat#4403087
Custom TaqMan SNP Genotyping Assay	ThermoFisher Scientific	Cat#4332077
KOD DNA polymerase	Merck Millipore	Cat#71086-4
GTXpress Taqman	ThermoFisher Scientific	Cat#4401892
Lipofectamine 2000 Transfection Reagent	ThermoFisher Scientific	Cat#11668019
RNeasy Mini Kit	Qiagen	Cat#74104
RNase-Free DNase	Qiagen	Cat#79254
QIAshredder	Qiagen	Cat#79654
TissueRuptor	Qiagen	Cat#9001271
NEBNext Ultra II DNA Library Prep with Sample Purification Beads	New England Biolabs	Cat#E7103S
NEBNext Multiplex Oligos for Illumina (Index Primers Set 1)	New England Biolabs	Cat#E7335S
Agilent High Sensitivity DNA Kit	Agilent	Cat#5067-4626
Agilent RNA 6000 Nano Kit	Agilent	Cat#5067-1511
Agilent RNA 6000 Nano Ladder	Agilent	Cat#5067-1529
NanoDrop 2000	ThermoFisher Scientific	Cat#ND-2000
2100-Bioanalyzer	Agilent	Cat#G2939BA
Illumina TotalPrep RNA Amplification Kit	Illumina	Cat#AMIL1791
MouseWG-6 v2.0 Expression BeadChip Kit	Illumina	Cat#BD-201-0202
**Strains**		
Apc^{Min}	Doug Winton	Sup Reference 10
Apc^{1322T}	Doug Winton	Sup Reference 11
Bcl9/Bcl9l DKO (*$Bcl9^{LoxP/LoxP}$*,*$B9l^{LoxP/LoxP}$*, *villin.Cre*/+)	Michel Aguet	Sup Reference 12
Pygo1/2 DKO (*$Pygo1^{-/-}$*, *$Pygo2^{LoxP/LoxP}$*, *villin.Cre*/+)	Michel Aguet	unpublished

Supplementary References

1. Bienz, M. & Hamada, F. Adenomatous polyposis coli proteins and cell adhesion. *Curr Opin Cell Biol* **16**, 528-535, doi:10.1016/j.ceb.2004.08.001 (2004).

2. Labbé, E. et al. Transcriptional cooperation between the transforming growth factor-beta and Wnt pathways in mammary and intestinal tumorigenesis. Cancer Res **11**, 75-85, doi:10.1158/0008-5472.CAN-06-2559 (2007).

3. Willert, J. et al. A transcriptional response to Wnt protein in human embryonic carcinoma cells. *BMC Dev Biol* **2**, 8, doi:10.1186/1471-213X-2-8 (2002).

4. Sansom, O. J. et al. Loss of Apc in vivo immediately perturbs Wnt signaling, differentiation, and migration. *Genes Dev* **18**, 1385-1390, doi:10.1101/gad.287404 (2004).

5. van der Flier, L. G. et al. The Intestinal Wnt/TCF Signature. *Gastroenterology* **132**, 628-632, doi:10.1053/j.gastro.2006.08.039 (2007).

6. Merlos-Suarez, A. et al. The intestinal stem cell signature identifies colorectal cancer stem cells and predicts disease relapse. *Cell Stem Cell* **8**, 511-524, doi:10.1016/j.stem.2011.02.020 (2011).

7. Munoz, J. et al. The Lgr5 intestinal stem cell signature: robust expression of proposed quiescent '+4' cell markers. *EMBO J* **31**, 3079-3091, doi:10.1038/emboj.2012.166 (2012).

8. van der Flier, L. G. et al. Transcription factor achaete scute-like 2 controls intestinal stem cell fate. *Cell* **136**, 903-912, doi:10.1016/j.cell.2009.01.031 (2009).

9. Lewis, A. *et al.* Severe polyposis in Apc(1322T) mice is associated with submaximal Wnt signalling and increased expression of the stem cell marker Lgr5. *Gut* **59**, 1680-1686, doi:10.1136/gut.2009.193680 (2010).

10. Moser, A. R., Pitot, H. C. & Dove, W. F. A dominant mutation that predisposes to multiple intestinal neoplasia in the mouse. *Science* **247**, 322-324 (1990).

11. Pollard, P. et al. The Apc 1322T mouse develops severe polyposis associated with submaximal nuclear beta-catenin expression. *Gastroenterology* **136**, 2204-2213 e2201-2213, doi:10.1053/j.gastro.2009.02.058 (2009).

12. Deka, J. et al. Bcl9/Bcl9l are critical for Wnt-mediated regulation of stem cell traits in colon epithelium and adenocarcinomas. *Cancer Res* **70**, 6619-6628, doi:10.1158/0008-5472.CAN-10-0148 (2010).
